# Multistate Switching of Spin Selectivity in Electron Transport through Light‐Driven Molecular Motors

**DOI:** 10.1002/advs.202101773

**Published:** 2021-07-22

**Authors:** Qirong Zhu, Wojciech Danowski, Amit Kumar Mondal, Francesco Tassinari, Carlijn L. F. van Beek, G. Henrieke Heideman, Kakali Santra, Sidney R. Cohen, Ben L. Feringa, Ron Naaman

**Affiliations:** ^1^ Department of Chemical and Biological Physics Weizmann Institute of Science Rehovot 76100 Israel; ^2^ Centre for Systems Chemistry Stratingh Institute for Chemistry University of Groningen Nijenborgh 4 Groningen 9747 AG The Netherlands; ^3^ Chemical Research Support Weizmann Institute of Science Rehovot 76100 Israel

**Keywords:** CISS effect, helix inversion, magnetic‐conductive atomic force microscope, molecular motor, spin polarization

## Abstract

It is established that electron transmission through chiral molecules depends on the electron's spin. This phenomenon, termed the chiral‐induced spin selectivity (CISS), effect has been observed in chiral molecules, supramolecular structures, polymers, and metal‐organic films. Which spin is preferred in the transmission depends on the handedness of the system and the tunneling direction of the electrons. Molecular motors based on overcrowded alkenes show multiple inversions of helical chirality under light irradiation and thermal relaxation. The authors found here multistate switching of spin selectivity in electron transfer through first generation molecular motors based on the four accessible distinct helical configurations, measured by magnetic‐conductive atomic force microscopy. It is shown that the helical state dictates the molecular organization on the surface. The efficient spin polarization observed in the photostationary state of the right‐handed motor coupled with the modulation of spin selectivity through the controlled sequence of helical states, opens opportunities to tune spin selectivity on‐demand with high spatio‐temporal precision. An energetic analysis correlates the spin injection barrier with the extent of spin polarization.

## Graphical abstract

The efficiency of electrons transport through chiral systems depends on their spin. Hence, chiral molecules are spin filters. Controlling the molecules' chirality provides a way to define the spin polarization. This molecular machine performs unidirectional rotations, inverting handedness of the molecule and therefore the spin‐polarization at each stage of the rotary cycle.

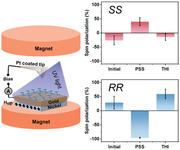

## 1 Introduction

The discovery of spin‐selective electron transport through chiral molecules, that is the chiral induced spin selectivity (CISS) effect, has laid a foundation for the development and construction of spin filters based entirely on organic materials.^[^ ^1^, ^2^, ^3^ ^]^ Thus far, the CISS effect was observed in a wide range of helically chiral biological and synthetic organic molecules including oligopeptides,^[^ ^4^ ^]^ DNA,^[^ ^5^ ^]^ helicenes,^[^ ^6^ ^]^ and more recently in helical covalent^[^ ^7^ ^]^ and supramolecular polymers.^[^ ^8^, ^9^ ^]^ However, to date most of these organic spin‐filters are based on molecules with fixed handedness, therefore limiting the spin of the transported electrons to that defined by the molecular chirality.^[^ ^2^ ^]^ A major challenge is the development of switchable organic spin‐filters, capable of inversion of the spin polarization. This would allow for the construction of responsive spintronic devices and therefore represents a major step forward in the field of organic spintronics.

Overcrowded‐alkene based molecular motors comprise an exceptional class of molecular machines capable of performing unidirectional rotary motion fueled by light and heat stimuli.^[^ ^10^, ^11^ ^]^ The rotary cycle of a typical motor comprises alternating photochemical and thermal isomerization steps, each involving inversion of the overall helical handedness of the molecule.^[^ ^12^ ^]^ Due to this unique chirality‐controlled motion, these molecules were exploited in a wide range of applications including photo‐switchable catalysts,^[^ ^13^, ^14^ ^]^ responsive liquid crystals,^[^ ^15^, ^16^, ^17^, ^18^ ^]^ and interfaces,^[^ ^19^, ^20^, ^21^ ^]^ biological systems^[^ ^22^ ^]^ and other hard^[^ ^23^, ^24^ ^]^ and soft materials.^[^ ^25^, ^26^ ^]^ Recently, studies on a donor‐acceptor functionalized second generation molecular motor^[^ ^27^ ^]^ revealed flipping of spin polarization of the tunnelled electrons upon inversion of motor handedness associated with the light‐induced isomerization.^[^ ^19^ ^]^

**Figure 1:**
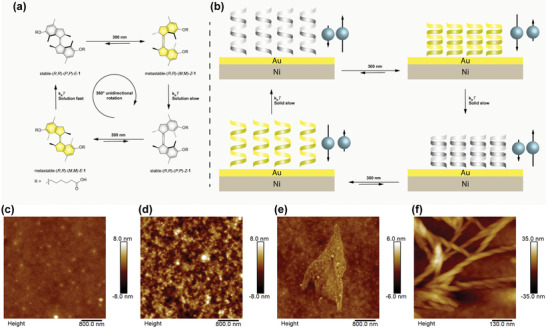
a) Molecular structure of helical diastereoisomers and rotary cycle of the 1st generation molecular motor **1**. The four‐step rotary cycle comprises two photochemical isomerizations (induced by 300 nm light) each followed by a thermal helix inversion. The stererochemical descriptors used, e.g., (*R*,*R*)‐(*P*,*P*)‐*E*‐**1** denote in order of appearance point chirality at the stereogenic centres, helical chirality and configuration of the double bond. b) Schematic depiction of the four‐stage spin polarization switching in electron tunneling through the molecular motor **1** thin film on nickel/gold (Ni/Au) substrate which is corresponding to unidirectional rotation through four helical states shown in a). There are four helical structures involved (right‐handed *P*,*P*; left‐handed *M*,*M*; right‐handed *P*,*P*; left‐handed *M*,*M* going through the 4‐step cycle as shown in a) dictating the change in chirality; the chiral molecules are depicted as helices. AFM topographies of the molecules drop casted on Ni/Au substrates are shown in c) (*S*,*S*)‐(*M*,*M*)‐*E*‐**1**, d) (*S*,*S*)‐(*M*,*M*)‐*Z*‐**1**. e) (*R*,*R*)‐(*P*,*P*)‐*E*‐**1** and f) (*R*,*R*)‐(*P*,*P*)‐*Z*‐**1**. Please note the higher resolution of f).

In this work, we demonstrate the first‐generation molecular motor scaffold acting as a light‐reconfigurable spin filter (**Figure** **1**).^[^ ^28^ ^]^ In contrast to their second‐generation counterparts, these compounds have four distinct chiral states that can be accessed in sequential manner in response to light and heat stimuli. Comparison of Raman spectroscopy data, acquired on drop‐casted and solution samples, demonstrated that these molecular motors undergo light and heat induced isomerization while deposited as thin films on gold substrates. The uniqueness of this system is that four states with distinct helical configurations can be interconverted non‐invasively with specific sequence (clockwise or counter‐clockwise). In addition, the carboxylic acid functionalities present a convenient handle for further functionalization and thus development of more sophisticated single‐molecule based switchable spin‐filters (Figure 1). The CISS magnetic‐conductive atomic force microscope (mc‐AFM) measurements indicated that these motors act as reconfigurable spin filters capable of inversion of the CISS effect upon each isomerization step. Importantly, despite characteristically moderate ratio of the metastable to stable isomers in the photostationary state for this scaffold, the spin‐polarization achieved in the irradiated samples represents nearly quantitative conversion to the (*R,R*)‐(*M,M*)‐*E* diastereoisomer.

## 2 Results

### 2.1 Morphology of Self‐Assembled Molecular Motor

The first‐generation molecular motor used in this study was designed based on a *p*‐xylene‐derived motor core, as the *E*‐ and *Z*‐ geometrical isomers of this overcrowded alkene scaffold can be conveniently obtained in enantiopure form on a large scale by co‐crystallization with a chiral resolving agent (see Figure 1a for structures).^[^ ^29^ ^]^ The four step unidirectional rotary cycle comprising alternating photochemical and thermal helix inversion steps sequentially converting **(*P*,*P*)‐*E*‐1** (right‐handed helix) to **(*M*,*M*)‐*Z*‐1** (left‐handed helix), to **(*P*,*P*)‐*Z*‐1** (right‐handed helix), to **(*M*,*M*)‐*E*‐1** (left‐handed helix) is shown in Figure 1a. Figure 1b depicts four‐stage spin polarization of thin films on Ni/Au substrates. The gold thin layer (8 nm thick) is protecting the Ni from oxidation while not interrupting the spin polarization.^[^ ^30^ ^]^ For thin films on Ni/Au substrates, AFM morphologies of (*S*,*S*)‐(*M*,*M*)‐*E*‐**1** (Figure 1c) and (*S*,*S*)‐(*M*,*M*)‐*Z*‐**1** (Figure 1d) display random aggregation. In the case of (*R*,*R*)‐(*P*,*P*)‐*E*‐**1** (Figure 1e), a µm‐sized layered structure is formed with thickness of 2–3 nm. In Figure 1f, the image of (*R*,*R*)‐(*P*,*P*)‐*Z*‐**1** reveals formation of chiral nanofibers.

### 2.2 The Rotary Motion of Molecular Motors in Solution

**Figure 2:**
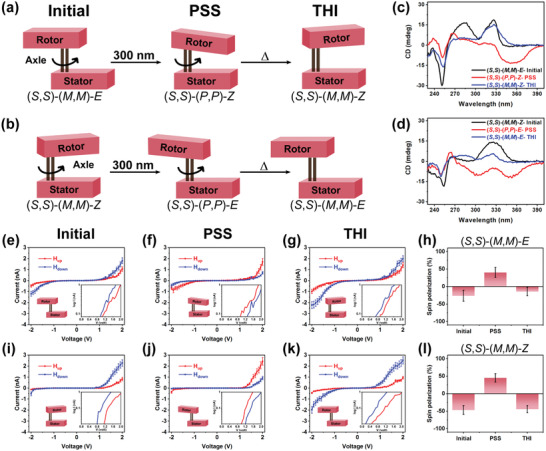
Rotary motion around the double‐bond axis of the first‐generation molecular motors. a) from (*S*,*S*)‐(*M*,*M*)‐*E* (Initial configuration) to (*S*,*S*)‐(*P*,*P*)‐*Z* (PSS) and then (*S*,*S*)‐(*M*,*M*)‐*Z* (THI); b) from (*S*,*S*)‐(*M*,*M*)‐*Z* (Initial configuration) to (*S*,*S*)‐(*P*,*P*)‐*E* (PSS) and then (*S*,*S*)‐(*M*,*M*)‐*E* (THI). Changes in the CD spectra obtained in tetrahydrofuran (THF) upon consecutive light and heat induced isomerization starting from c) (*S*,*S*)‐(*M*,*M*)‐*E*‐**1** and d) (*S*,*S*)‐(*M*,*M*)‐*Z*‐**1**. Averaged *I*–*V* curves of (*S*,*S*)‐(*M*,*M*)‐*E*‐**1** and (*S*,*S*)‐(*M*,*M*)‐*Z*‐**1** under both directions of magnetic field. For (*S*,*S*)‐(*M*,*M*)‐*E*‐**1**, the three measured conditions are e) initial (*S*,*S*)‐(*M*,*M*)‐*E*‐**1**, f) irradiated (*S*,*S*)‐(*P*,*P*)‐*Z*‐**1** (PSS), and g) reversed (*S*,*S*)‐(*M*,*M*)‐*Z*‐**1** (THI). For (*S*,*S*)‐(*M*,*M*)‐*Z*‐**1**, the corresponding conditions are i) initial (*S*,*S*)‐(*M*,*M*)‐*Z*‐**1**, j) irradiated (*S*,*S*)‐(*P*,*P*)‐*E*‐**1** (PSS), and k) reversed (*S*,*S*)‐(*M*,*M*)‐*E*‐**1** (THI). Panels h) and l) are histograms summarizing the spin polarization of (*S*,*S*)‐(*M*,*M*)‐*E*‐**1** and (*S*,*S*)‐(*M*,*M*)‐*Z*‐**1** at 2 V. Error bars represent the standard errors. The insets are semilog plots of the data. H_up_ corresponds to north pole of the magnet pointing up while H_down_ is the opposite polarity.

**Figure 3:**
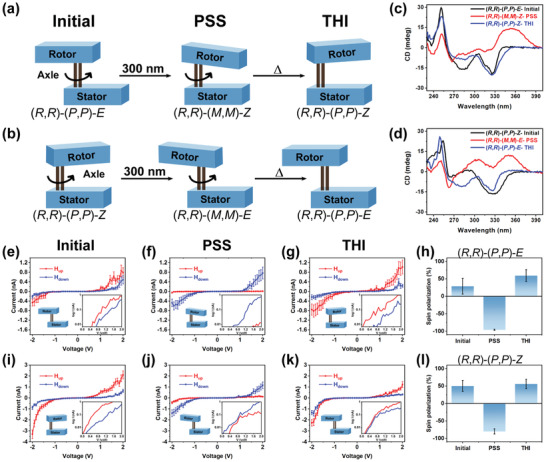
Rotary motion around a double‐bond axis of first‐generation molecular motors: a) from (*R*,*R*)‐(*P*,*P*)‐*E* (Initial configuration) to (*R*,*R*)‐(*M*,*M*)‐*Z* (PSS) and then (*R*,*R*)‐(*P*,*P*)‐*Z* (THI); b) from (*R*,*R*)‐(*P*,*P*)‐*Z* (initial configuration) to (*R*,*R*)‐(*M*,*M*)‐*E* (PSS) and then (*R*,*R*)‐(*P*,*P*)‐*E* (THI). Changes in the CD spectra (THF) upon consecutive light and heat induced isomerization starting from c) (*R*,*R*)‐(*P*,*P*)‐*E*‐**1** and d) (*R*,*R*)‐(*P*,*P*)‐*Z*‐**1**. Averaged *I*–*V* curves of (*R*,*R*)‐(*P*,*P*)‐*E*‐**1** and (*R*,*R*)‐(*P*,*P*)‐*Z*‐**1** under both directions of magnetic field. For (*R*,*R*)‐(*P*,*P*)‐*E*‐**1**, the three measured conditions are e) initial (*R*,*R*)‐(*P*,*P*)‐*E*‐**1**, f) irradiated (*R*,*R*)‐(*M*,*M*)‐*Z*‐**1** (PSS), and g) reversed (*R*,*R*)‐(*P*,*P*)‐*Z*‐**1** (THI). For (*R*,*R*)‐(*P*,*P*)‐*Z*‐**1**, the corresponding conditions are i) initial (*R*,*R*)‐(*P*,*P*)‐*Z*‐**1**, j) irradiated (*R*,*R*)‐(*M*,*M*)‐*E*‐**1** (PSS), and k) reversed (*R*,*R*)‐(*P*,*P*)‐*E*‐**1** (THI). Panels h) and l) are histograms summarizing the spin polarizations at 2 V. Error bars represent the standard errors. The insets are semilog plots of the data.

Combined UV/Vis absorption, CD (Figures S18–S21, Supporting Information), Raman (Figures S25–S27, Supporting Information), and ^1^H NMR (Figures S12–S24, Supporting Information) spectroscopy data indicated that the designed motor operates in solution in essentially the same manner as the parent structure.^[^ ^29^ ^]^ Starting from either stable (*R*,*R*)‐(*P*,*P*)‐*E*‐**1** or (*R*,*R*)‐(*P*,*P*)‐*Z*‐**1** isomers (Figure 1a) a unidirectional rotary cycle (clockwise), involving 4 states, is observed for these 1st generation motors. (Note that starting from left‐handed helical isomers the same sequence of isomerizations is followed but in the counter‐clockwise rotary cycle). Irradiation of either *E*‐ or *Z*‐isomers at 300 nm, resulted in the inversion of the handedness of the molecule (see CD spectra, **Figures** **2**c,d and **3**c,d; and Figures S18 and S21, Supporting Information) leading to the formation of the metastable *Z*‐**1** (from *E* isomer) or metastable *E*‐**1** isomers (from *Z* isomer), (75:25 of metastable *Z*‐**1**:stable *E*‐**1**, 71:29 of metastable *E*‐**1**:stable *Z*‐**1**, Figures S23 and S22, Supporting Information). These species subsequently undergo a thermal isomerization (thermal helix inversion, THI) to the respective stable isomers through a right‐handed (Figure 1a) thermal helix inversion Δ^‡^ *G* (20°C) = 100.1 ± 0.4 kJ mol^−1^, *t*_1/2_ = 21 h for metastable *Z*‐**1** to stable *Z*‐**1** isomerization, and Δ^‡^ *G* (20 °C) = 78 ± 6 kJ mol^−1^, *t*_1/2_ = 10 s for metastable *E*‐**1** to stable *E*‐**1** isomerization (Figures S21 and S19, Supporting Information). At room temperature in solution only three states of the rotary cycle could be observed, due to the low barrier of the thermal helix inversion of the *E*‐metastable diastereomer, thus effectively making the motor **1** a three‐state chiroptical switch (Figures S24 and S27, Supporting Information).

### 2.3 Raman Studies in Solid State

Raman micro‐spectroscopy data recorded on the molecular motor drop‐casted on gold substrate (Figures S28 and S29, Supporting Information) indicated that irradiation of both stable *E*‐**1** and stable *Z*‐**1** isomers at 300 nm leads to the formation of the respective metastable isomers, i.e., metastable *Z*‐**1** and metastable *E*‐**1**. This could readily be identified by the Raman shifts of the bands characteristic of the stretching of the central olefinic bond, thus demonstrating that both compounds can undergo light‐induced isomerization in the solid (Figures S30 and S32, Supporting Information). However, upon prolonged exposure of the stable *E*‐**1** isomer to 300 nm light, some degree of decomposition was observed, most probably associated with the photopolymerization of the overcrowded alkene, while the stable *Z*‐**1** isomer showed much higher photostability. Importantly, in contrast to the solution species, when dropcasted on the surface the activation barriers of the thermal helix inversion of metastable *Z*‐**1** and metastable *E*‐**1** isomers were found to be similar (*t*_1/2_ ≈1 h), as derived from Raman data recorded with a 500 mW 785 nm laser (Figures S31 and S33, Supporting Information). While the observed enhanced rate of thermal helix inversion for the metastable *Z*‐**1** to stable *Z*‐**1** isomer can be attributed to local heating caused by the high‐power Raman laser, the remarkable increase in thermal stability of the metastable *E*‐**1** isomer most probably originates from the intermolecular interactions between the tightly packed molecules in the drop‐casted film. The degree of heating caused by the Raman laser should in both cases be similar. Therefore, the comparable thermal stability of the metastable *Z*‐**1** and *E*‐**1** isomers suggest that both processes proceed in the solid state with an activation Gibbs free energy of at least Δ^‡^ *G* (20 °C) > 100 kJ mol^−1^, which is the barrier for the metastable *Z*‐**1** to stable *Z*‐**1** isomerization in solution. This demonstrates that, motor **1** drop‐casted on a gold substrate operates as four state light‐ and heat‐reconfigurable chiroptical switch.

We observed that in addition to the change in the molecular conformation upon exposure to UV light and heat, distinct organization of the molecules adsorbed on the surface (Figure 1c–f). This is especially evident in the case of (*R*,*R*)‐(*P*,*P*)‐*E*‐**1** and (*R*,*R*)‐(*P*,*P*)‐*Z*‐**1**: the first forms a close packed layer and the second forms chiral nanofibers.

### 2.4 Magnetic‐Conductive Atomic Force Microscopy Studies in Solid State

Figure 2 presents the current versus voltage (*I‐V*) curves obtained in the mc‐AFM measurements on films of left‐handed helical molecules (*S*,*S*)‐(*M*,*M*)‐*E* and (*S*,*S*)‐(*M*,*M*)‐*Z* (Figure 2a,b) deposited on a silicon substrate covered with a gold‐coated nickel film (Ni/Au 120/8 nm thickness). Each curve represents an average of about 80 individual spectra. The AFM was positioned inside an electromagnet allowing in‐situ substrate magnetization with the magnetic pole pointing up (*H*_up_, red curves) or down (*H*_down_, blue curves) along the sample normal. The applied magnetic field was 0.5 T. The insets present the positive quadrant of the *I‐V* curve in a semi‐log plot. Figure 2e–g are results following the photo‐induced isomerization of (*S*,*S*)‐(*M*,*M*)‐*E*‐**1**. In the initial (*S*,*S*)‐(*M*,*M*)‐*E*‐**1** (Figure 2a), the averaged current magnitude measured with the magnetic field pointing down is higher than for magnetic field pointing up for all nonzero voltages (Figure 2e). The PSS obtained upon irradiation has opposite chirality (Figure 2a), leading to reversal in the observed spin selectivity. Therefore, Figure 2f shows higher current for *H*_up_. However, (*S*,*S*)‐(*P*,*P*)‐*Z*‐**1** (PSS) (Figure 2a) is metastable and undergoes thermal isomerization to (*S*,*S*)‐(*M*,*M*)‐*Z*‐**1** (THI) after a few days, as evidenced by reversal of the spin selectivity to the original preference for *H*_down_ (Figure 2g). For these solid‐state films at ambient conditions, each step in the molecular motor rotary cycle is much slower than in solution. We choose the data at 2 V to compare the spin polarization (SP) for all spectra. The spin polarization is defined as *SP* (%)  = (*I*_UP_ − *I*_DOWN_)/(*I*_UP_ + *I*_DOWN_) × 100  when *I*_UP_ and *I*_DOWN_ are the currents measured for *H*_up_ or *H*_down_, respectively. The SP data obtained from Figure 2e–g are summarized in the histogram (Figure 2h). The sequential modulation using (*S*,*S*)‐(*M*,*M*)‐*E* from negative to positive and then negative spin polarization is associated with light and heat induced changes in the helicity of the deposited motors from (*M*,*M*) to (*P*,*P*) and (*M*,*M*) during the three step process constituting half of the rotary cycle (Figure 2h).

To complete the cycle, the study was continued starting from pure (*S*,*S*)‐(*M*,*M*)‐*Z*‐**1** isomer (Figure 2b) in order to exclude interference from other diastereomers present in the photostationary state mixture. Figure 2i–k present the averaged *I‐V* curves of the unperturbed (*S*,*S*)‐(*M*,*M*)‐*Z*‐**1**, (*S*,*S*)‐(*P*,*P*)‐*E*‐**1** (PSS) and of (*S*,*S*)‐(*M*,*M*)‐*E*‐**1** (THI), which show similar spin selectivity as was observed for the curves shown in Figure 2e–g, reflecting the helicity change in each isomerization step (for histograms see Figure 2l). **Table** **1** presents the observed SP for all the studied configurations. The SP of the PSS configuration does not depend on the initial species, (*S*,*S*)‐(*M*,*M*)‐*E*‐**1** or (*S*,*S*)‐(*M*,*M*)‐*Z*‐**1**, and the sign of the SP is identical for each configuration (initial, PSS, or THI) for the both molecules, showing that the helicity, either (*M*,*M*) or (*P*,*P*) dictates the sign of SP. We stress that the transformation from trans (*E*‐**1**) to cis (*Z*‐**1**) isomers with the same inherent handedness of the electronic *π*‐system (*M*,*M*‐helicity) does not affect the spin selectivity. A unique feature of the motor based system is that starting with, e.g., enantiomer (*R*,*R*)‐(*P*,*P*)‐**1** a four‐step right‐handed (clockwise) rotary cycle is followed. This implies precise control over the sequence of the helicity change and consequently modulation of the spin polarization.

**Table 1:** Summary of the SP at 2 V for initial, PSS and THI of molecular motors: (*S*,*S*)‐(*M*,*M*)‐*E*‐**1**, (*S*,*S*)‐(*M*,*M*)‐*Z*‐**1**, (*R*,*R*)‐(*P*,*P*)‐*E*‐**1**, and (*R*,*R*)‐(*P*,*P*)‐*Z*‐**1**

Molecules	Initial [%]	PSS [%]	THI [%]
(*S*,*S*)‐(*M*,*M*)‐*E*‐**1**	−26 ± 16	40 ± 14	−14 ± 12
(*S*,*S*)‐(*M*,*M*)‐*Z*‐**1**	−47 ± 13	45 ± 12	−44 ± 10
(*R*,*R*)‐(*P*,*P*)‐*E*‐**1**	29 ± 23	−96 ± 2	60 ± 17
(*R*,*R*)‐(*P*,*P*)‐*Z*‐**1**	50 ± 16	−80 ± 7	56 ± 14

The insets of Figure 2e–g,i–k indicate that the current under each spin condition has a different voltage threshold, i.e., a different spin injection barrier (see **Table** **2**). This voltage difference (H_up_ and H_down_) comparing initial configuration, PSS and THI for (*S*,*S*)‐(*M*,*M*)‐*E*‐**1** are +0.1, −0.3, and +0.2 V, respectively. For (*S*,*S*)‐(*M*,*M*)‐*Z*‐**1**, these differences are +0.4, −0.2, and +0.3 V. These values are large compared to thermal energy at room temperature and similar to values obtained in other CISS studies.^[^ ^31^ ^]^ They are consistent with the significant spin polarization observed at room temperature.

The spin selective *I*–*V* curves for the stable isomers with (*P*,*P*) helicity, i.e., (*R*,*R*)‐(*P*,*P*)‐*E*‐**1** (Figure 3a) and (*R*,*R*)‐(*P*,*P*)‐*Z*‐**1** (Figure 3b) are presented in Figure 3e–g,i–k. The spin selectivity observed for these species is opposite to that observed for the corresponding stable left‐handed (*M*,*M*) isomers, shown in Figure 2e–g, i.e., the spin selectivity for (*R*,*R*)‐(*P*,*P*)‐*Z*‐**1** is opposite to that observed for the corresponding isomers of the configuration (*S*,*S*)‐(*M*,*M*)‐*Z*‐**1**. The changes in the sign of SP (Figure 3h,l) accompanying the isomerization of motor **1** starting from either (*R*,*R*)‐(*P*,*P*)‐*E*‐**1** or (*R*,*R*)‐(*P*,*P*)‐*Z*‐**1** follow the sequence positive to negative to positive, that is again reflecting the modulation of molecular helicity from (*P*,*P*) to (*M*,*M*) to (*P*,*P*), in accordance with the changes in the CD spectra (Figure 3c,d). It is important to note that, while in principle the form of the conduction through all molecules is remarkably similar, some differences in spin selectivity, as well as asymmetry in conduction for positive or negative bias are observed in all the samples.

## 3 Discussion

As summarized in Table 1, the absolute spin selectivity observed for the isomers of **1** at the PSS and THI of both (*R*,*R*)‐(*P*,*P*)‐*E*‐**1** and (*R*,*R*)‐(*P*,*P*)‐*Z*‐**1**, is higher than for (*S*,*S*)‐(*M*,*M*)‐*E*‐**1** and (*S*,*S*)‐(*M*,*M*)‐*Z*‐**1**. Suda et al. studied *M*‐helical second‐generation molecular motor species, finding their structure significantly different from the system studied here.^[^ ^19^ ^]^ Their reported value of the SP for the initial state is 43% at 1.9 V and 44% for the PSS. These SP values are very similar to our results on stable *M*‐helical motors. Table 2 summarizes the differences in the spin injection barriers for the two spins for all studied configurations. The values hover around a few hundred meV except for the PSS of (*R*,*R*)‐(*P*,*P*)‐*E*‐**1**. Indeed, in this configuration also the spin polarization is by far the largest and is 96% (Table 1).

**Table 2:** Summary of the spin injection barriers for initial, PSS, and THI of molecular motors: (*S*,*S*)‐(*M*,*M*)‐*E*‐**1**, (*S*,*S*)‐(*M*,*M*)‐*Z*‐**1**, (*R*,*R*)‐(*P*,*P*)‐*E*‐**1**, and (*R*,*R*)‐(*P*,*P*)‐*Z*‐**1**

Molecules	Initial ± 0.03 [V]	PSS ± 0.03 [V]	THI ± 0.03 [V]
(*S*,*S*)‐(*M*,*M*)‐*E*‐**1**	0.1	−0.3	0.2
(*S*,*S*)‐(*M*,*M*)‐*Z*‐**1**	0.4	−0.2	0.3
(*R*,*R*)‐(*P*,*P*)‐*E*‐**1**	−0.2	1.0	−0.5
(*R*,*R*)‐(*P*,*P*)‐*Z*‐**1**	−0.2	0.3	−0.1

The present experiments reveal the effect of configurational change on the electron conduction and spin transport properties. There are several observations that may hint at the structure‐function relation in the studied systems. While the *I‐V* curves for the Initial and PSS configurations of both (*S*,*S*)‐(*M*,*M*)‐*E*‐**1** and (*S*,*S*)‐(*P*,*P*)‐*Z*‐**1** systems are highly asymmetric, namely the current is much higher when the bias is positive than for negative biases (see Figure 2), it is symmetric for the THI configuration. Since, in principle, the THI state should be identical to the initial configuration in terms of molecular handedness (the THI of (*S*,*S*)‐(*M*,*M*)‐*E*‐**1** is identical to the Initial (*S*,*S*)‐(*M*,*M*)‐*Z*‐**1** and vice versa) the asymmetry in the THI must originate from effects unrelated to the helicity of the molecules, for example supramolecular arrangement of the molecules on the substrate in both Initial and PSS states. Our data unequivocally show that given the proper supramolecular arrangement of the molecular motors on the substrates, nearly quantitative SP in the tunneled electrons can be achieved within these reconfigurable molecular machines.

For these experiments made on a film deposited on a surface, it is impossible to assess the role of intermolecular interaction on the results, namely to what extent the results presented relate to conduction through a single molecule or through some supramolecular structure that could consist of molecules forming *π*–*π* interactions. Our topographic AFM measurements clearly indicate a dramatic change in the organization of the molecules on the surface, as a result of changing the molecular configuration. For these drop‐casted samples, the molecular orientation on the surface is not well defined, although likely at some of the helical molecules are oriented parallel to the surface, to favor the interactions of the *π*‐system with the substrate. It is remarkable that, despite all these variables, the spin filtering correlates so well with the changes in the solution CD spectra. The configuration, as well as the sequence of formation of the helical structures – and as a consequence the sign of SP – can be precisely controlled noninvasively with light. These findings indicate that the mechanism of the CISS effect depends on the intrinsic properties of the molecules and not on the details of the conduction path. Such properties include polarizability and its anisotropy and most notable the intrinsic helicity.^[^ ^32^ ^]^

## 4 Conclusion

In conclusion, this work presents the dependence of the spin selective conduction on structural changes of the molecular motors as a result of photoisomerization (initial and PSS) followed by thermal relaxation (THI). Remarkably high spin selectivity was observed in the photostationary state mixture of isomers obtained by photoisomerization, as compared to the initial structures. Our results clearly show that each of the four states of the unidirectional rotary cycle is characterized by distinct differences in SP and the system can be used in multistage control over modulation of spin polarization with high sequence‐specificity. The data presented here provide guidelines for designing highly efficient molecular spin filters based on the helical overcrowded alkene structures, in which the photo‐generated isomers (PSS) are stabilized against thermal relaxation.

## 5 Experimental Section

### CD Studies

For the CD studies, solutions of tetrahydrofuran (THF) were degassed by sparging with Argon for ≈1 min in a 1 cm quartz cuvette equipped with the septum‐sealed screw cap. Starting from both *E* **‐1**‐stable diastereomers, samples were irradiated at 312 nm at room temperature until no further changes were observed in the UV–vis spectrum and kept overnight in the dark at 40 °C to complete thermal isomerization. Starting from both *Z* **‐1**‐stable diastereomers, samples were irradiated at 312 nm at −20 °C and the progress was monitored using UV–vis absorption spectroscopy. Upon reaching photostationary state samples were quickly moved to a precooled (−15 °C) CD sample holder and a CD spectrum was recorded. For the thermal helix inversion samples were warmed to room temperature and kept in the dark for ≈2 h to ensure complete conversion.

### Raman Studies

For the Raman studies in the solid, solutions of either *E*‐**1** or *Z*‐**1** (0.1 mg mL^−1^) were prepared in THF drop‐casted on gold substrates and left to dry under air. As prepared samples were irradiated on a microscope stage at 300 nm using M300F2 LED guided to the sample by optical fiber. Irradiations were carried for ≈3–4 min. for *Z*‐**1** and ≈10 min. for *E*‐**1**. Spectra were processed with simple and adaptive baselines, normalized and averaged using Spectragryph software.

### Magnetic Conductive Probe Atomic Force Microscopy (mc‐AFM) Studies

Substrates were made by sputtering a 120 nm layer of nickel, followed by an 8 nm layer of gold on top of a silicon wafer with a 2 µm thermal silicon oxide layer, with a 10 nm titanium adhesion layer. All surfaces were cleaned by first immersing in boiling acetone and then in ethanol for 10 min, followed by UV‐ozone cleaning for 15 min and a final incubation in warm ethanol for 40 min. 1mg mL^−1^ molecular motor was dissolved in dry tetrahydrofuran (THF), drop‐casted onto the substrate and dried in a nitrogen glove box under dark. Deep UV (300 nm) Fiber‐Coupled LED, SMA, 350 mA, 320 µW (Min) was used to irradiate the samples for 10 min in a nitrogen glove box. AFM topographies were obtained by Bruker‐AFM MultiMode 8 with a silicon tip (Model AC240TS‐R3 from Oxford instruments). Magnetic field‐dependent *I*–*V* measurements were obtained with the probe in contact with the surface with a controlled force (8–10 nN). Cantilever B (nominal force constant 2.7 N m^−1^) of a platinum‐coated silicon tip from Micromasch (HQ:DPE‐XSC11) was used in the measurements. For each state, ≈80–100 *I*–*V* curves were recorded with a constant applied magnetic field of 0.5 T for each magnetic field orientation (field UP(H_up_) and DOWN (H_down_)). These curves were plotted as a graph by OriginPro and analyzed by average multiple curves function.

## Conflict of Interest

The authors declare no conflict of interest.

## Author Contributions

Q.R.Z., W.D., and A.K.M. contributed equally to this work. W.D. synthesized molecules and performed CD and Raman studies. W.D. and C.L.F.B performed UV/Vis and NMR studies. Q.R.Z, F.T., G.H.H., and K.S. involved in sample preparation. Q.R.Z. and A.K.M. performed mc‐AFM measurements. Q.R.Z. and S.C. analyzed the data. Q.R.Z., S.C. and R.N and B.L.F. wrote the manuscript. B.L.F and R.N. conceived the experiments and guided the project.

## Supporting information

Supporting Information

## Data Availability

The data that support the findings of this study are available from the corresponding author upon reasonable request.
