# Supplementary material for: Multistate Switching of Spin Selectivity in Electron Transport through Light‐Driven Molecular Motors
Source: Adv Sci (Weinh). 2021 Jul 22;8(18):2101773. doi: 10.1002/advs.202101773 (PMC8456272; doi:10.1002/advs.202101773)
Supplement: Supplementary file 1 — Supporting Information [file ADVS-8-2101773-s001.pdf]

© 2021 Wiley-VCH GmbH

ADVANCED SCIENCE Open Access

Supporting Information

for *Adv. Sci.,* DOI: 10.1002/advs.202101773

Multistate Switching of Spin Selectivity in Electron Transport Through light – Driven Molecular Motors

Qirong Zhu, Wojciech Danowski, Amit Kumar Mondal, Francesco Tassinari, Carlijn L. F. van Beek, G. Henrieke Heideman, Kakali Santra, Sidney R. Cohen, Ben L. Feringa,* and Ron Naaman **

Supplemental Information

Multistate switching of spin selectivity in electron transport through light – driven molecular motors

Qirong Zhu, Wojciech Danowski, Amit Kumar Mondal, Francesco Tassinari, Carlijn L. F. van Beek, G. Henrieke Heideman, Kakali Santra, Sidney R. Cohen, Ben L. Feringa,* and Ron Naaman **

1. General remarks

All reagents were obtained from commercial sources and used as received without further purification. Dry solvents were obtained from an MBraun solvent purification system. Column chromatography was performed on a Reveleris X2 flash chromatography system. TLC: silica gel 60, Merck, 0.25 mm. Molecular motor **3** was synthesized according to a literature procedure.[1] The enantiomers of **3** were separated following previously described protocol.[1] HRMS were recorded on an LTQ Orbitrap XL. NMR spectra were obtained using a Varian Mercury Plus (^{1}H: 400 MHz, ^{13}C: 100 MHz). Chemical shifts are reported in δ units (ppm) relative to the residual solvent signal of $CDCl_3$ (^{1}H NMR, δ 7.26 ppm; ^{13}C NMR, δ 77.0 ppm), THF-d_8 (^{1}H NMR, δ 1.72 ppm, 3.58 ppm) or MeOH-d_4 (^{1}H NMR, δ 3.31 ppm). The splitting pattern is designated as follows: s (singlet), d (doublet), t (triplet), m (multiplet), br (broad), p (quintet), dd (doublet of doublets) ddd (doublet of doublets of doublets). Chiral HPLC analysis was performed using HPLC system (LC 10ADVP, Shimadzu) equipped with a diode array detector (SPDM10AVP, Shimadzu) using a Lux Cellulose columns with mixtures of HPLC grade heptane and 2-propanol as eluent and column temperature of 40°C. Sample injections were made using a HP 6890 Series Auto sample Injector. UV/Vis absorption spectra were measured on diode array spectrometers (8453, Agilent or Specord S-600, Analytik Jena AG) equipped with Peltier cuvette holder (Luma 40/Univ-Short, Quantum Northwest) and temperature controller (TC1, Quantum Northwest) in 1 cm quartz cuvettes. CD spectra were recorded on a spectrometer (J-810 CD, Jasco) equipped with Peltier device (PFD-425, Jasco) in 1 cm cuvettes. THF used for spectroscopic studies was obtained from an MBraun solvent purification system. Irradiation experiments were performed using Thorlab LEDs (M365FP1, λ_{max} = 365 nm and M300L4 or M300F2 λ_{max} = 300 nm) or Spectroline lamp (ENB-280C/FE, λ_{max} = 312 nm). Raman spectra in solution were recorded on home-built system comprising sample holder with magnetic stirrer, 785 nm 400 mW laser (Cobolt, 08-NLDM) guided through a Raman probe and connected to spectrograph (Kymera 193i, Andor TM Technology) equipped with CCD camera (iDus 416, Andor TM Technology). Raman spectra at room and elevated temperature were recorded using cuvette holder (Luma 40/Univ-Short, Quantum Northwest) equipped with temperature controller (TC1, Quantum Northwest) and at low temperature using cryogenic temperature controlled sample holder (CoolSpeK UV USP-203, Unisoku). Raman spectra in solid were were recorded on home-built system comprising microscope (BX-50F-3, Olympus), 785 nm 500 mW diode laser (BT-785-PLR500-FCPC-1, Coherent) connected to spectrograph (Shamrock, Andor TM Technology) equipped with CCD camera (iVac 316, Andor TM Technology). CISS measurements were performed using an atmospheric electro-magnet AFM system consisting of a Besocke-Beetle inertial Ambient AFM controlled by R9 electronics (RHK Technology) housed in an HV-7V electromagnet (Laboratoria Elettrofisico Walker LDJ Scientific) with a continuously adjustable (up to 1T) magnetic field.

2. Experimental Procedures

UV/Vis absorption studies. For the UV/Vis studies, solutions (THF) were degassed by sparging with Argon for ca. 1 min in a 1 cm quartz cuvette equipped with the septum-sealed screw cap. For the kinetic studies of the thermal helix inversion the solutions were irradiated at λ_{max} = 312 nm at temperatures of (-16.0 °C to -20.0 °C, every 1.0 °C for (±)-***E*-1**-metatable to (±)-***E*-1**-stable thermal helix inversion or 45.0 °C to 55.0 °C, every 2.5 °C for (±)-***Z*-1**-metastable to (±)-***Z*-1**-stable thermal helix inversion) until no further changes were observed in the UV/Vis absorption spectrum. Afterwards the irradiation was discontinued and the changes in absorbance were followed in time. All the spectra were opened and processed with Spectragryph software using simple baseline tool.

Rate constants at respective temperatures were extracted by fitting monoexponential decay function to the data using Origin software. Eyring plots were constructed from the data to extrapolate the Gibbs free energy of activation of thermal helix inversion to room temperature. Uncertainty in Gibbs free energy of activation was estimated as 95 % confidence interval extrapolated to room temperature.

^{1}H NMR studies. For ^{1}H NMR studies, solution (MeOH-*d*$_4$) were degassed by sparging with Ar for ca. 30 s in the quartz NMR tube or by three cycles of freeze-pump-thaw cycles by freezing the solution at -115 °C in an ethanol/liquid N_2 bath. Starting from (±)-***E*-1**-stable sample was irradiated at λ_{max} = 312 nm at -5 °C until the photostationary state was reached and subsequently kept over night in the dark at 40 °C to complete thermal isomerization. Starting from (±)-***Z*-1**-stable sample was irradiated at λ_{max} = 312 nm at -60 °C in a cryostat cooling bath (ethanol) and placed in a pre-cooled spectrometer for the measurements. Next the sample was allowed to warm to room temperature and kept in the dark for ca. 10 min. to complete thermal helix inversion.

Raman studies in solution For the Raman studies, solutions of motors *E*-**1** or *Z*-**1** (ca. 8 mg/mL) were prepared in absolute methanol. The solution were degassed by sparging with Argon for ca. 1 min in a 1 cm quartz cuvette equipped with the septum-sealed screw cap and stirring bar. Solutions were irradiated at 300 nm (M300L4 Throlabs LED) at appropriate temperature (RT or -50 ºC) with vigorous stirring until no further changes were observed in Raman spectrum. Spectra were processed with simple and adaptive baselines, normalized and averaged using Spectragryph software.

3. Synthetic procedures and Chromatograms

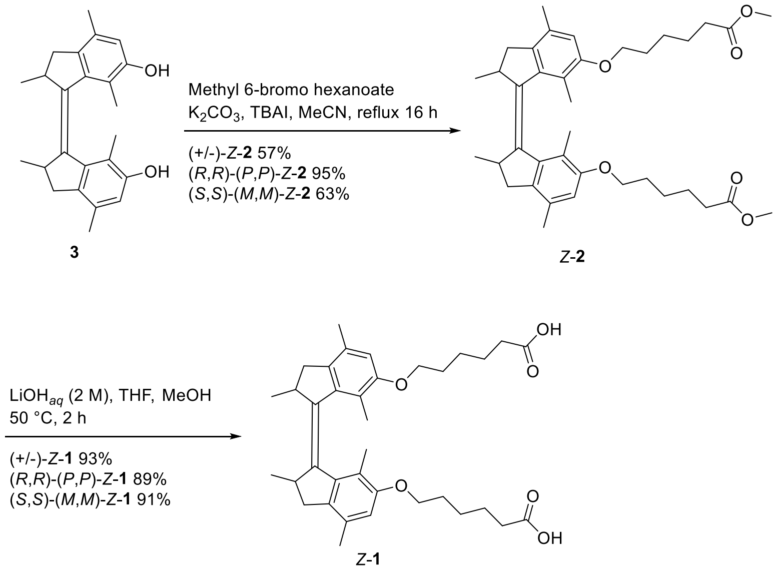

Scheme S1. Synthesis of molecular motor *Z*-**1.**

Dimethyl 6,6'-((2,2',4,4',7,7'-hexamethyl-2,2',3,3'-tetrahydro-[1,1'-biindenylidene]-6,6'-diyl)bis(oxy)) (*Z*)-dihexanoate (*Z*-2).

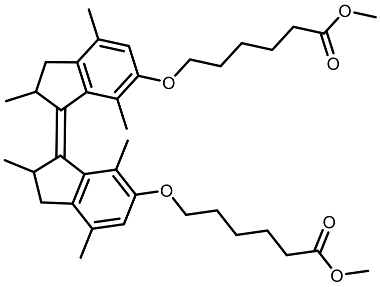

A mixture of **3** (200 mg, 0.57 mmol, 1.0 equiv.), methyl-6-bromohexanoate (477 mg, 2.28 mmol, 4.0 equiv.), K_2CO_3 (473 mg, 3.42 mmol, 6.0 equiv.), tetrabutylammonium iodide (22 mg, 0.06 mmol, 0.1 equiv.) and acetonitrile (25 mL) was stirred at reflux for 20 h. The reaction mixture was diluted with EtOAc (100 mL), washed with water (3 x 30 mL), and brine (2 x 75 mL) and dried over anhydrous $MgSO_4$. The crude product was concentrated *in vacuo* and purified by column chromatography (SiO_2, pentane:EtOAc) to yield (±)-***Z*-2** as a colourless oil solidifying upon standing (200 mg, 0.33 mmol, 58%). (±)-*Z*-**2**: Rf = 0.22 (pentane:EtOAc 9:1). ^1H NMR (400 MHz, $CDCl_3$) δ 6.51 (s, 2H), 4.05 – 3.94 (m, 2H), 3.88 – 3.76 (m, 2H), 3.65 (s, 6H), 3.32 (p, *J* = 6.4 Hz, 2H), 3.03 (dd, *J* = 14.6, 6.4 Hz, 2H), 2.37 (d, *J* = 14.6 Hz, 2H), 2.32 (t, *J* = 7.5 Hz, 4H), 2.24 (s, 6H), 1.82 – 1.72 (m, 4H), 1.68 (p, *J* = 7.8 Hz, 4H), 1.53 – 1.42 (m, 4H), 1.37 (s, 6H), 1.06 (d, *J* = 6.7 Hz, 6H).^{13}C NMR (100 MHz, $CDCl_3$) δ 174.3, 155.9, 142.3, 141.1, 136.1, 130.5, 122.6, 111.8, 68.5, 51.6, 42.0, 38.2, 34.2, 29.4, 25.9, 24.9, 20.6, 19.0, 14.4. (ESI^+) calcd for $C_{38}H_{53}O_6$ $[M+H]^+$ 605.3837, found 605.3829.

(*R*,*R*)-(*P*,*P*)-*Z*-**2** was synthesized following a similar general procedure from (*R*,*R*)-(*P*,*P*)-**3** (200 mg, 0.57 mmol, 1.0 equiv.), methyl-6-bromohexanoate (477 mg, 2.28 mmol, 4.0 equiv.), K_2CO_3 (473 mg, 3.42 mmol, 6.0 equiv.), tetrabutylammonium iodide (22 mg, 0.06 mmol, 0.1 equiv.) and acetonitrile (15 mL) to yield 330 mg (0.54 mmol, 95%) of product. HPLC (Luxcellulose-1, 0.5 mL/min., heptane:IPA 99:1) *t* = 24.3 min. *ee*>99%

(S,S)-(M,M)-Z-**2** was synthesized following similar general procedure from (S,S)-(M,M)-**3** (340 mg, 0.98 mmol, 1.0 equiv.), methyl-6-bromohexanoate (815 mg, 3.90 mmol, 4.0 equiv.), K_2CO_3 (808 mg, 5.85 mmol, 6.0 equiv.), tetrabutylammonium iodide (181 mg, 0.49 mmol, 0.5 equiv.) and acetonitrile (15 mL) to yield 370 mg (0.62 mmol, 63%) of product. HPLC (Luxcellulose-1, 0.5 mL/min., heptane:IPA 99:1) t = 20.9 min. *ee*>99%

(Z)-6,6'-((2,2',4,4',7,7'-hexamethyl-2,2',3,3'-tetrahydro-[1,1'-biindenylidene]-6,6'-diyl)bis(oxy)) dihexanoic acid (Z-1).

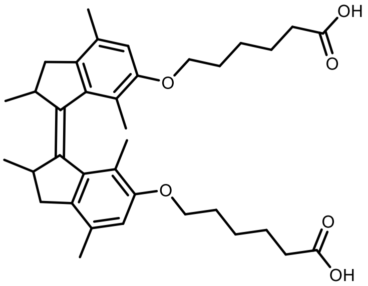

An aqueous solution of $LiOH_{aq}$ (2.0 M, 1.1 mL, 10.0 equiv.) was added to mixture of (±)-Z-**2** (140 mg, 0.23 mmol, 1.0 equiv.) in THF (5 mL) and MeOH (5 mL) and the reaction mixture was heated at 50 °C for 2 h. Subsequently the mixture was concentrated *in vacuo*, dissolved in water and quenched with aqueous HCl (1 M, 2.5 mL). Next, the mixture was extracted with EtOAc (3 x 20 mL) and the organic layer was washed with water (5 x 20 mL) and brine (2 x 20 mL), dried over $MgSO_4$ and subsequently the solvent was removed under reduced pressure. The resulting pale yellow solid was triturated with pentane/CH_2Cl_2 (15/1 v/v) and dried in *vacuo* to yield (±)-Z-**1** as white solid (124 mg, 0.21 mmol, 93 %). m. p. 141.6 – 142.6 ºC, ^{1}H NMR (400 MHz, $CDCl_3$) δ 6.55 (s, 2H), 4.07 – 3.93 (m, 2H), 3.93 – 3.78 (m, 2H), 3.32 (p, J = 6.5 Hz, 2H), 3.03 (dd, J = 14.5, 6.3 Hz, 2H), 2.46 – 2.32 (m, 6H), 2.24 (s, 6H), 1.83 – 1.64 (m, 8H), 1.61 – 1.46 (m, 4H), 1.38 (s, 6H), 1.26 (s, 2H), 1.06 (d, J = 6.7 Hz, 6H). ^{13}C NMR (100 MHz, $CDCl_3$) δ 179.6, 155.9, 142.3, 141.1, 136.5, 130.6, 122.9, 112.6, 69.0, 42.0, 38.2, 34.1, 29.5, 25.9, 24.6, 20.6, 18.9, 14.4. HMRS (ESI$^+$) calcd for $C_{36}H_{49}O_6$ $[M+H]^+$ 577.3524, found 577.3512.

(R,R)-(P,P)-Z-**1** was synthesized following similar general procedure from (R,R)-(P,P)-Z-**2** (150 mg, 0.25 mmol, 1.0 equiv.) and $LiOH_{aq}$ (2.0 M, 2.5 mL, 20.0 eqiv.) in THF (5 mL) and MeOH (5 mL) to yield 128 mg (0.22 mmol, 89%) of the product. m. p. 153.2 – 154.1 ºC

(S,S)-(M,M)-Z-**1** was synthesized following similar general procedure from (S,S)-(M,M)-Z-**2** (250 mg, 0.41 mmol, 1.0 equiv.) and $LiOH_{aq}$ (2.0 M, 4.1 mL, 20.0 eqiv.) in THF (5 mL) and MeOH (5 mL) to yield 215 mg (0.37 mmol, 91%) of the product. m. p. 153.5 – 154.5 ºC

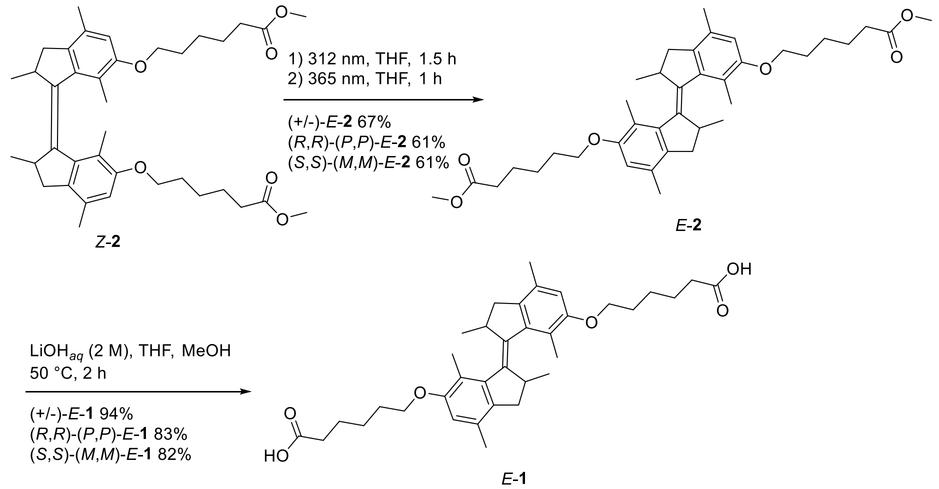

Scheme S2. Synthesis of molecular motor *E*-**1.**

Dimethyl 6,6'-2,2',4,4',7,7'-hexamethyl-2,2',3,3'-tetrahydro-[1,1'-biindenylidene]-6,6'-diyl)bis(oxy))(*E*)-dihexanoate (*E*-2).

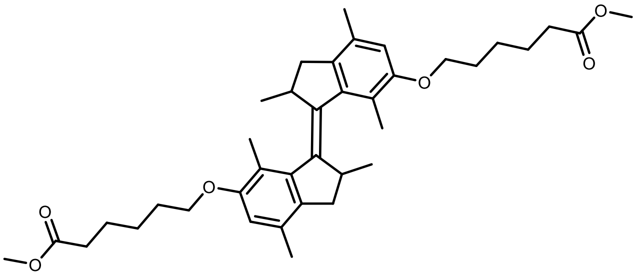

A 1 cm quartz cuvette was filled with a solution of (±)-*Z*-**2** (20.0 mg, 33.1 µmol) in 2.5 mL of THF, then degassed by purging with argon for ca. 30 s and subsequently tightly sealed with a septum equipped screw cap. Three such cuvettes were then placed between two UV lamps (ENB-280C/FE) ca. 2 cm from each lamp. The samples were then irradiated at room temperature first at 312 nm for 1 h and then at 365 nm for 1 h. The solutions were combined, concentrated under reduced pressure and purified by column chromatography (SiO_2, pentane:EtOAc) to yield (±)-*E*-**2** as a colourless oil which solidified upon standing (40.0 mg, 66.1 µmol, 67%). (±)-*E*-**2**: Rf = 0.31 (pentane:EtOAc 9:1). ^{1}H NMR (400 MHz, $CDCl_3$) δ 6.52 (s, 2H), 4.13 – 3.85 (m, 4H), 3.68 (s, 6H), 2.89 (p, *J* = 6.3 Hz, 2H), 2.59 (dd, *J* = 14.0, 5.5 Hz, 2H), 2.38 (t, *J* = 7.5 Hz, 4H), 2.29 (s, 6H), 2.17 (m, 8H), 1.85 (dt, *J* = 13.3, 6.7 Hz, 4H), 1.75 (p, *J* = 7.6 Hz, 4H), 1.63 – 1.55 (m, 4H), 1.09 (d, *J* = 6.4 Hz, 6H). ^{13}C NMR (100 MHz, $CDCl_3$) δ 174.3, 156.4, 142.6, 141.8, 134.1, 131.3, 120.6, 111.0, 68.1, 51.7, 42.3, 38.5, 34.2, 29.4, 26.0, 24.9, 19.4, 18.8, 16.3. HRMS (ESI$^+$) calcd for $C_{38}H_{53}O_6$ $[M+H]^+$ 605.3837, found 605.3824.

(*R*,*R*)-(*P*,*P*)-*E*-**2** was synthesized following similar general procedure from (*R*,*R*)-(*P*,*P*)-*Z*-**2** (150 mg, 0.25 mmol, 8 batches) to yield 92 mg (0.15 mmol, 61%) of product. HPLC (Luxcellulose-1, 0.5 mL/min., heptane:IPA 99.3:0.7) *t* = 37.6 min. *ee*>99%

(*S*,*S*)-(*M*,*M*)-*E*-**2** was synthesized following similar general procedure from (*S*,*S*)-(*M*,*M*)-*Z*-**2** (140 mg, 0.23 mmol, 7 batches), to yield 85 mg (0.14 mmol, 61%) of product. HPLC (Luxcellulose-1, 0.5 mL/min., heptane:IPA 99.3:0.7) *t* = 41.9.6 min. *ee*>99%

(*E*)-6,6'-((2,2',4,4',7,7'-hexamethyl-2,2',3,3'-tetrahydro-[1,1'-biindenylidene]-6,6'-diyl)bis(oxy)) dihexanoic acid (*E*-1).

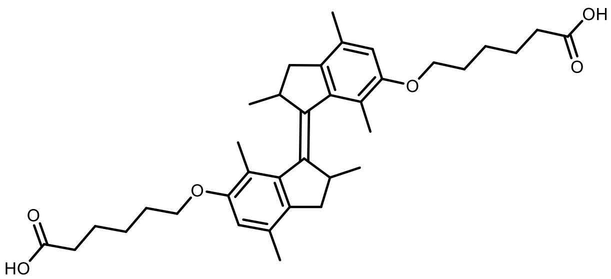

An aqueous solution of $LiOH_{aq}$ (2.0 M, 0.35 mL, 10.0 equiv.) was added to mixture of (±)-***E*-2** (40.0 mg, 0.066 mmol, 1.0 equiv.) in THF (2 mL) and MeOH (2 mL) and the reaction mixture was heated at 50 °C for 2 h. Subsequently the mixture was concentrated *in vacuo*, dissolved in water and quenched with aqueous HCl (1 M, 2.5 mL). Next, the mixture was extracted with EtOAc (3 x 20 mL) and the organic layer was washed with water (5 x 20 mL) and brine (2 x 20 mL), dried over $MgSO_4$ and subsequently the solvent was removed under reduced pressure. The resulting pale yellow solid was triturated with pentane/CH_2Cl_2 (15/1 v/v) and dried in *vacuo* to yield (±)-***E*-1** as white solid (36 mg, 0.062 mmol, 94 %). (±)-***Z*-1**: m. p. 170.1 – 171.1 ºC, ^{1}H NMR (400 MHz, $CDCl_3$) δ 6.52 (s, 2H), 4.11 – 3.88 (m, 4H), 2.89 (p, *J* = 6.3 Hz, 2H), 2.59 (dd, *J* = 14.1, 5.6 Hz, 2H), 2.43 (t, *J* = 7.4 Hz, 4H), 2.29 (s, 6H), 2.19 – 2.11 (m, 8H), 1.92 – 1.82 (m, 4H), 1.76 (p, *J* = 7.5 Hz, 4H), 1.66 – 1.56 (m, 4H), 1.08 (d, *J* = 6.4 Hz, 6H). ^{13}C NMR (100 MHz, $CDCl_3$) δ 178.7, 156.3, 142.6, 141.8, 134.1, 131.3, 120.6, 111.0, 68.1, 42.3, 38.5, 33.9, 29.4, 26.0, 24.7, 19.4, 18.8, 16.3. HMRS (ESI^+) calcd for $C_{36}H_{49}O_6$ $[M+H]^+$ 577.3524, found 577.3511.

(*R*,*R*)-(*P*,*P*)-***E*-1** was synthesized following similar general procedure from (*R*,*R*)-(*P*,*P*)-***E*-2** (90 mg, 0.15 mmol, 1.0 equiv.) and $LiOH_{aq}$ (2.0 M, 1.5 mL, 20 equiv.) in THF (5 mL) and MeOH (5 mL) to yield 71 mg (0.12 mmol, 83 %) of product. m. p. 165.6 – 166.4 ºC

(*S*,*S*)-(*M*,*M*)-***E*-1** was synthesized following similar general procedure from (*S*,*S*)-(*M*,*M*)-***E*-2** (80 mg, 0.13 mmol, 1.0 equiv.) and $LiOH_{aq}$ (2.0 M, 1.3 mL, 20 equiv.) in THF (5 mL) and MeOH (5 mL) to yield 63 mg (0.11 mmol, 82 %) of product. m. p. 165.8 – 166.4 ºC

CD spectra and HPLC traces

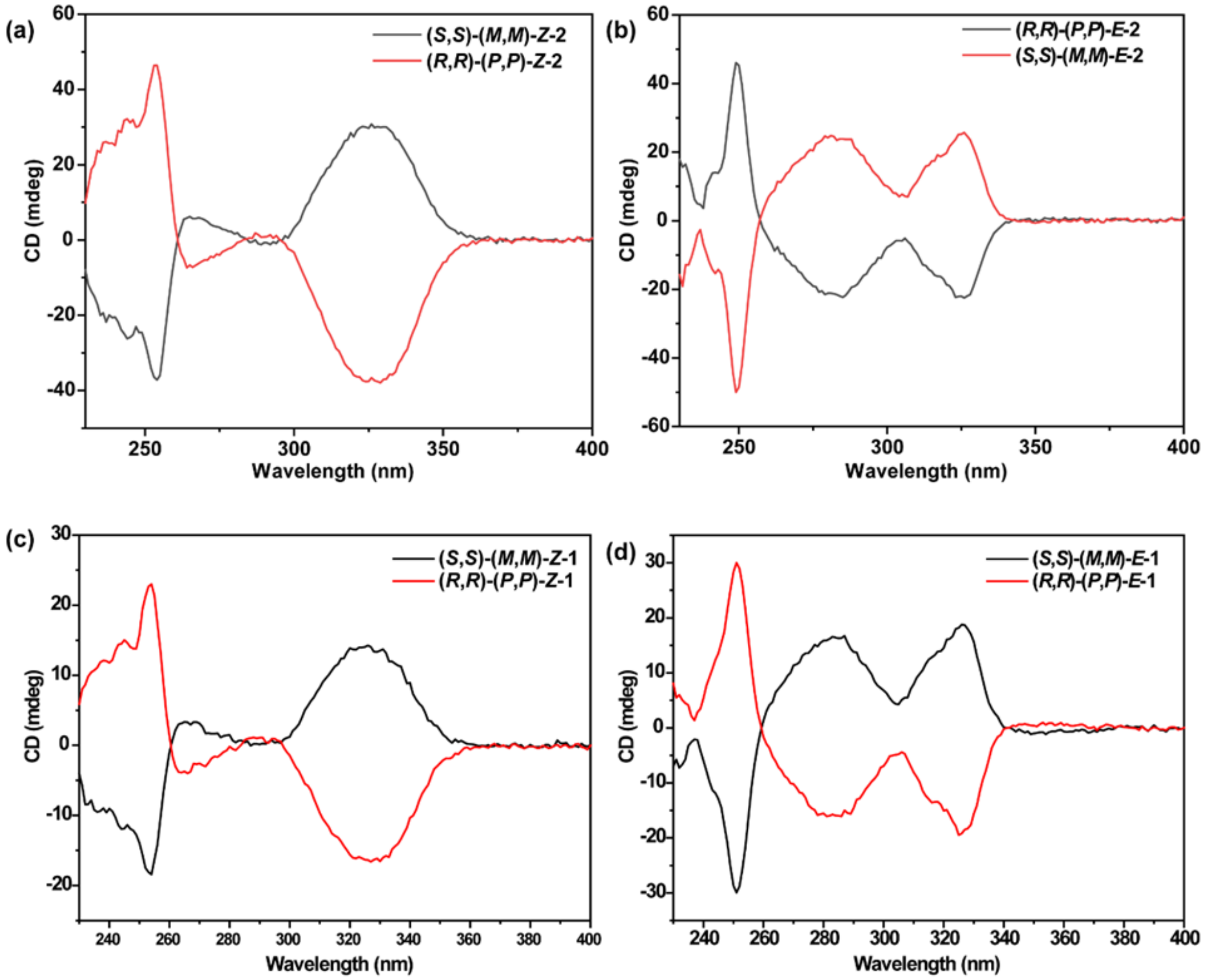

Figure S1. CD spectra (THF, RT) of (a) (*S*,*S*)-(*M*,*M*)-***Z*-2** (19.6 μM) and (*R*,*R*)-(*P*,*P*)-***Z*-2** (23.1 μM) (b) (*S*,*S*)-(*M*,*M*)-***E*-2** (26.1 μM) and (*R*,*R*)-(*P*,*P*)-***E*-2** (22.9 μM) (c) (*S*,*S*)-(*M*,*M*)-***Z*-1** (17.1 μM) and (*R*,*R*)-(*P*,*P*)-***Z*-1** (14.7 μM) (d) (*S*,*S*)-(*M*,*M*)-***E*-1** (15.3 μM) and (*R*,*R*)-(*P*,*P*)-***E*-1** (15.1 μM).

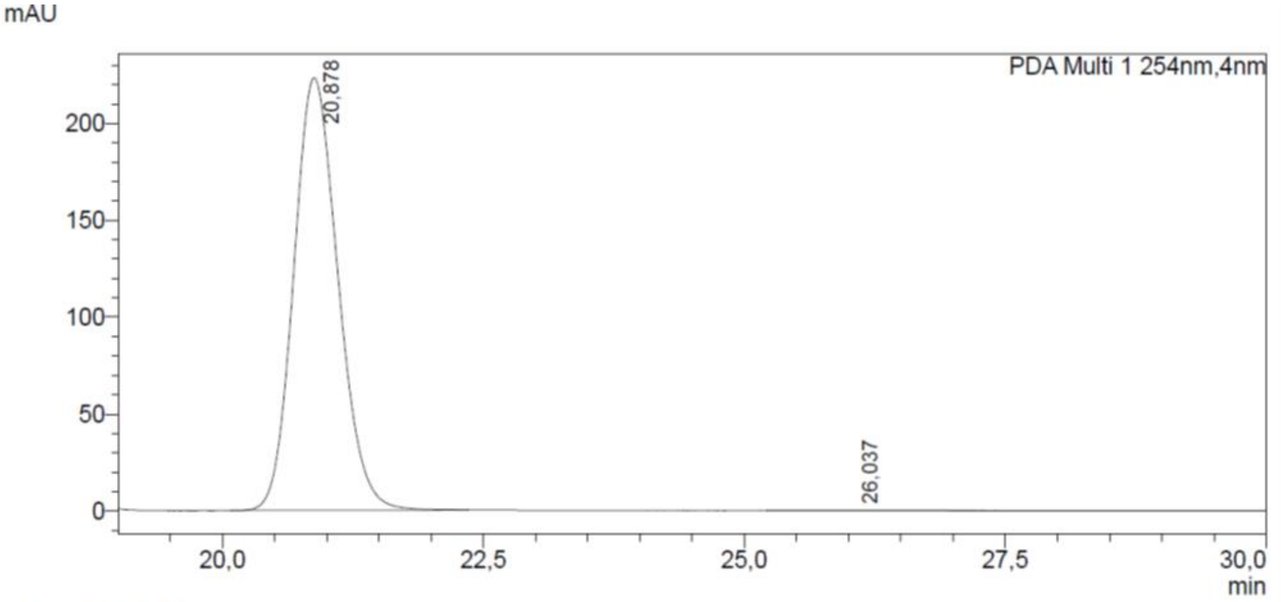

PDA Ch1 254nm

Peak#	Ret. Time	Area	Height	Conc.	Unit	Mark	Area%
1	20,878	6590675	223272	0,000			99,885
2	26,037	7579	127	0,000		M	0,115
Total		6598254	223399				100,000

Figure S2. HPLC trace (Luxcellulose-1, 0.5 mL/min., heptane:IPA 99:1, 40 ºC) of (*S*,*S*)-(*M*,*M*)-*Z*-**2**

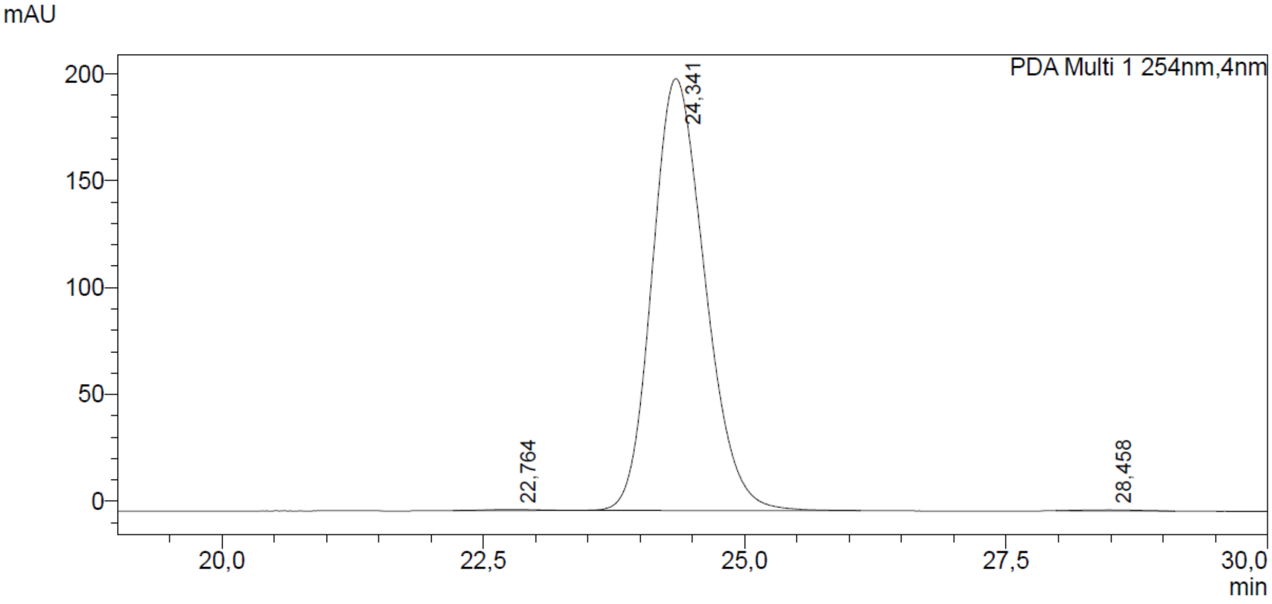

<Peak Table>

PDA Ch1 254nm

Peak#	Ret. Time	Area	Height	Conc.	Unit	Area%
1	5,937	2603	279	0,000		0,037
2	22,764	16940	529	0,000		0,238
3	24,341	7085925	202068	0,000		99,501
4	28,458	16024	459	0,000		0,225
Total		7121492	203336			100,000

Figure S3. HPLC trace (Luxcellulose-1, 0.5 mL/min., heptane:IPA 99:1, 40 ºC) of (*R*,*R*)-(*P*,*P*)-*Z*-**2**

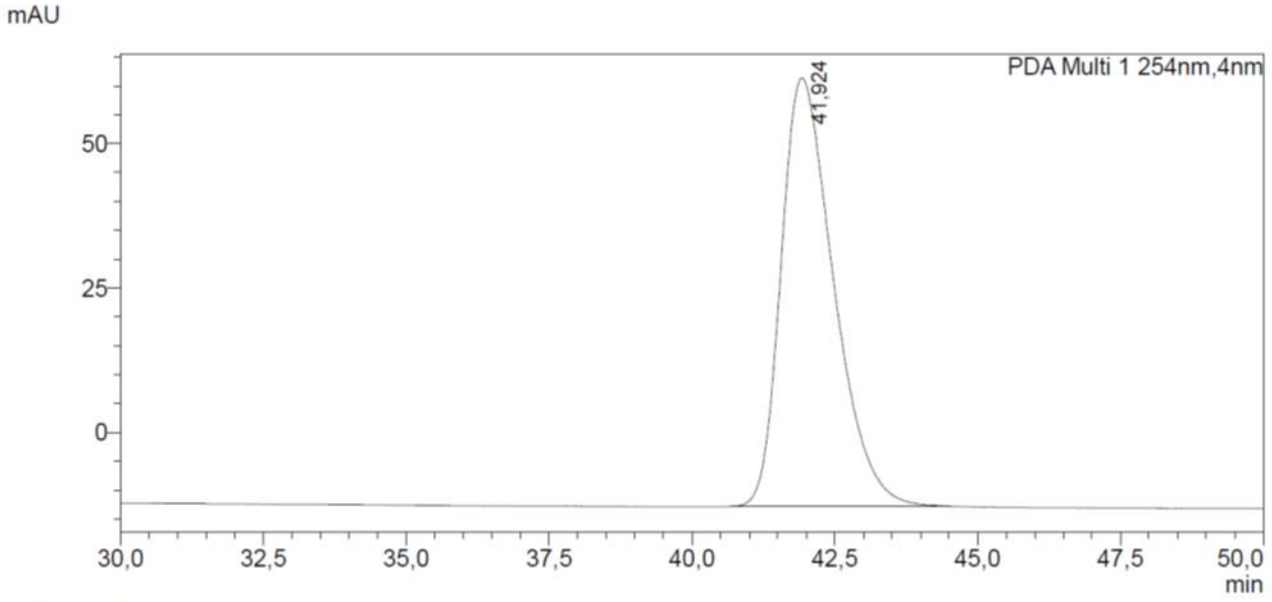

<Peak Table>

PDA Ch1 254nm

Peak#	Ret. Time	Area	Height	Conc.	Unit	Mark	Area%
1	41,924	4742014	74128	0,000			100,000
Total		4742014	74128				100,000

Figure S4. HPLC trace (Luxcellulose-1, 0.5 mL/min., heptane:IPA 99.3:0.7, 40 ºC) of (*S*,*S*)-(*M*,*M*)-*E*-**2**

<Chromatogram>

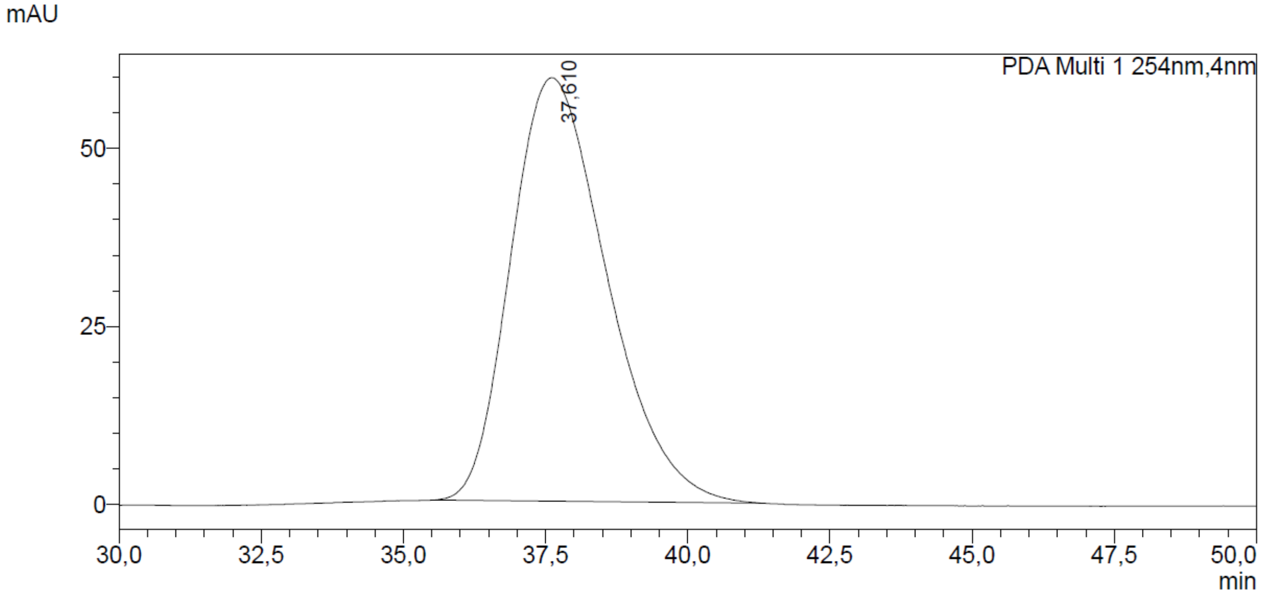

<Peak Table>

PDA Ch1 254nm

Peak#	Ret. Time	Area	Height	Conc.	Unit	Mark	Area%
1	37,610	6984116	59408	0,000			100,000
Total		6984116	59408				100,000

Figure S5. HPLC trace (Luxcellulose-1, 0.5 mL/min., heptane:IPA 99.3:0.7, 40 ºC) of (*R*,*R*)-(*P*,*P*)-*E*-**2**

^{1}H, ^{13}C NMR Spectra, HRMS Spectra of New Compounds

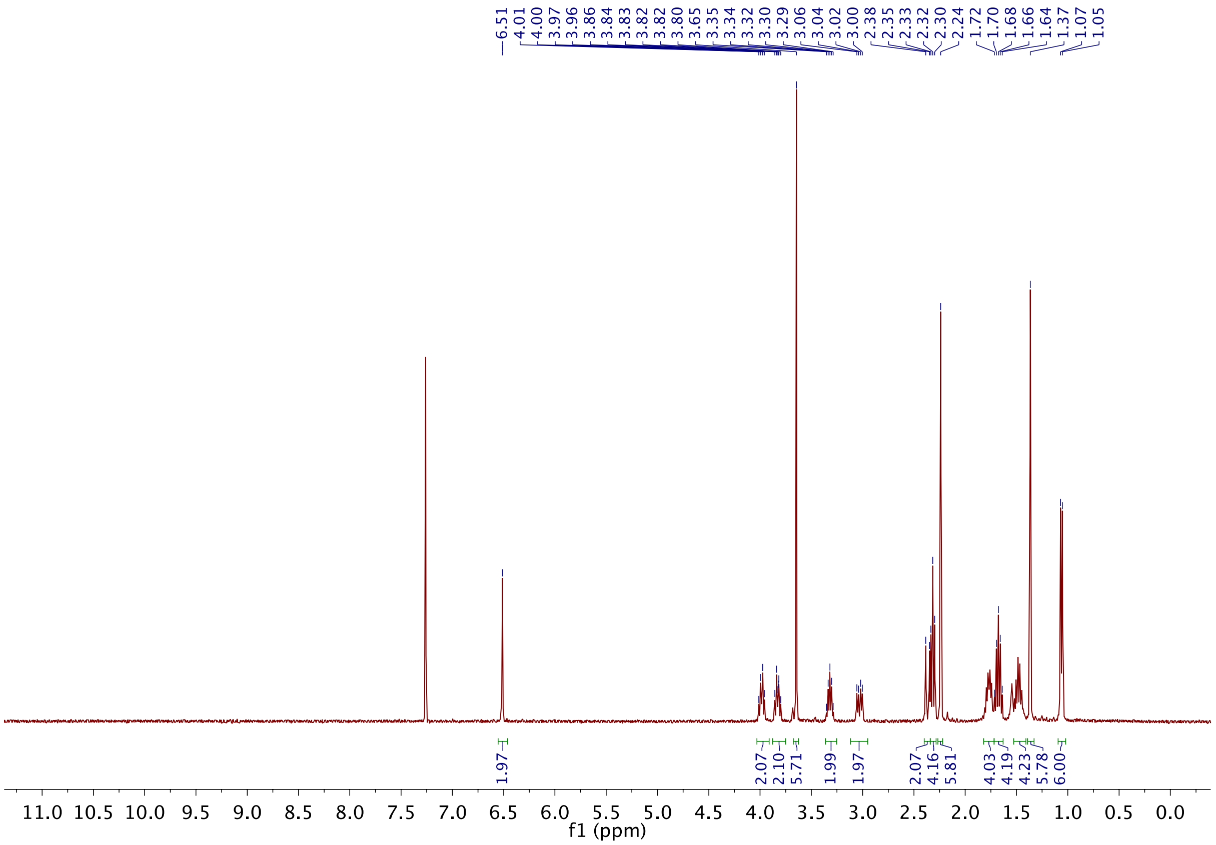

Figure S6. ^{1}H-NMR spectrum of ***Z*-2** ($CDCl_3$, 20 °C).

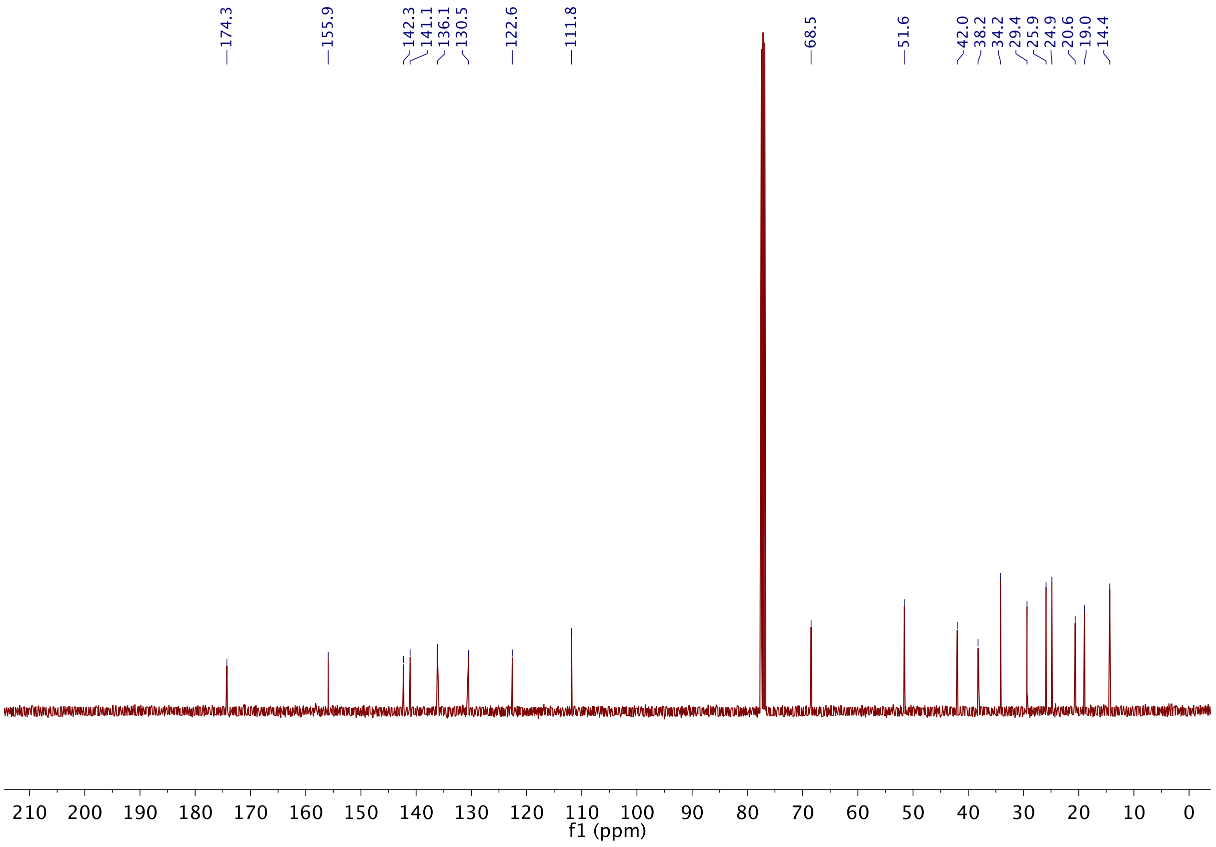

Figure S7. ^{13}C-NMR spectrum of ***Z*-2** ($CDCl_3$, 20 °C).

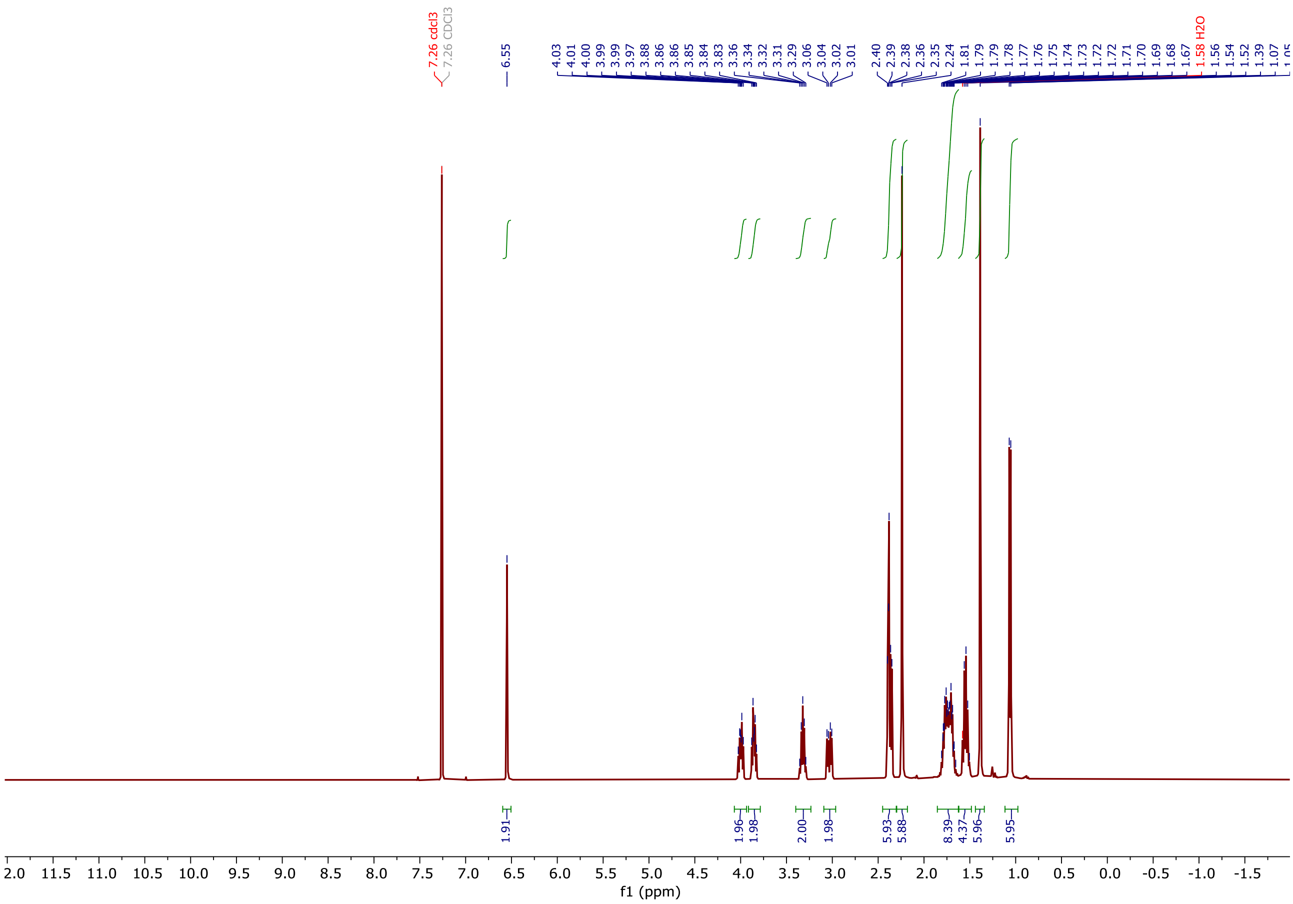

Figure S8. ^{1}H-NMR spectrum of ***Z*-1** ($CDCl_3$, 20 °C).

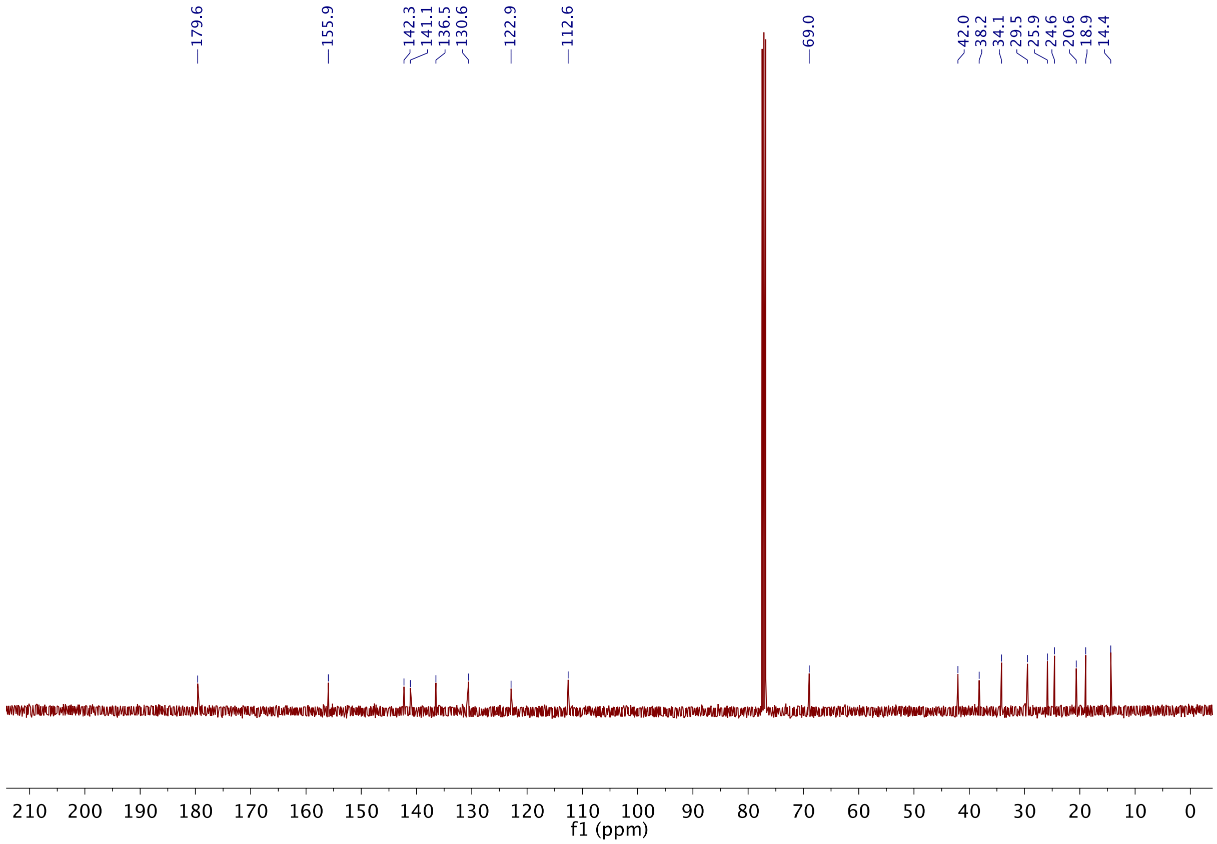

Figure S9. ^{13}C-NMR spectrum of ***Z*-1** ($CDCl_3$, 20 °C).

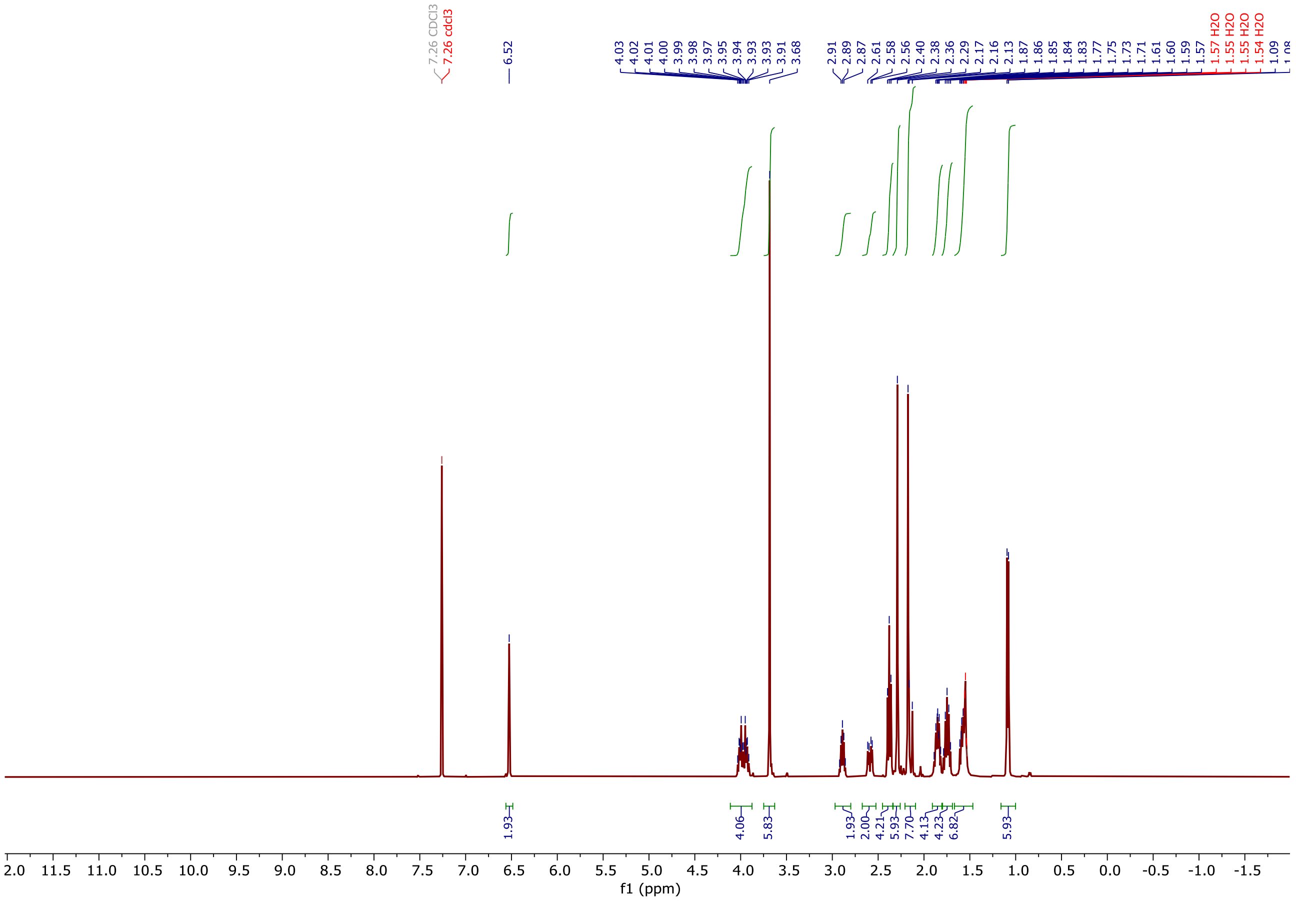

Figure S10. ^{1}H-NMR spectrum of ***E*-2** ($CDCl_3$, 20 °C).

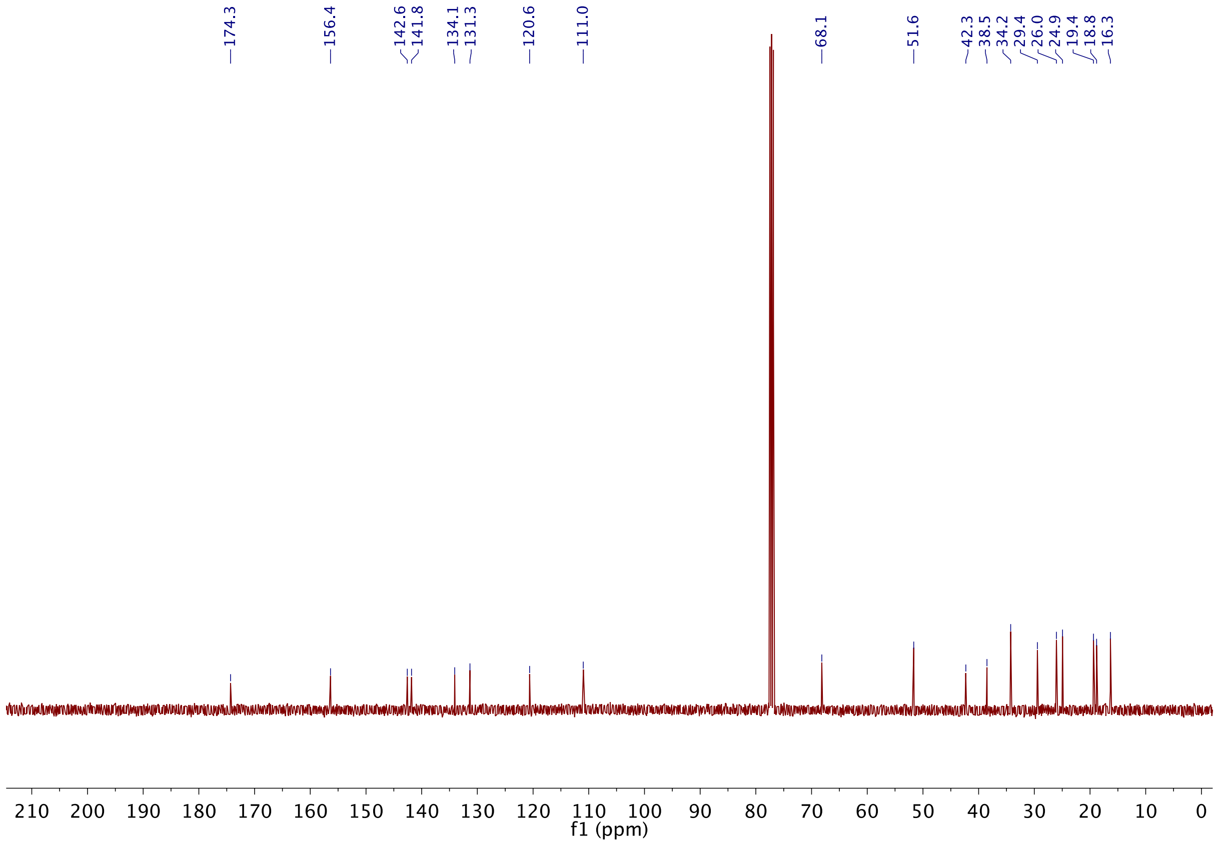

Figure S11. ^{13}C-NMR spectrum of ***E*-2** ($CDCl_3$, 20 °C).

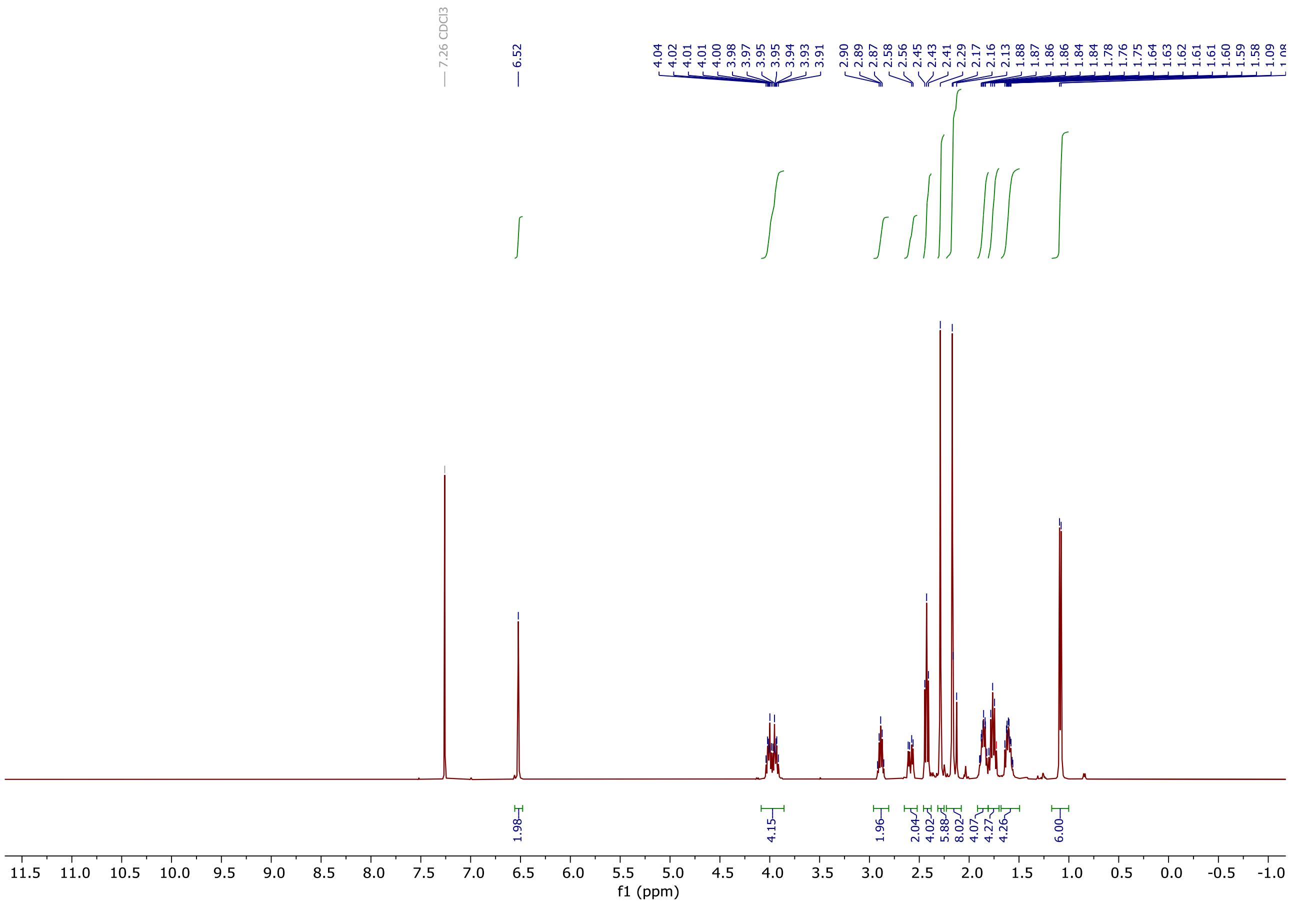

Figure S12. ^{1}H-NMR spectrum of ***E*-1** ($CDCl_3$, 20 °C).

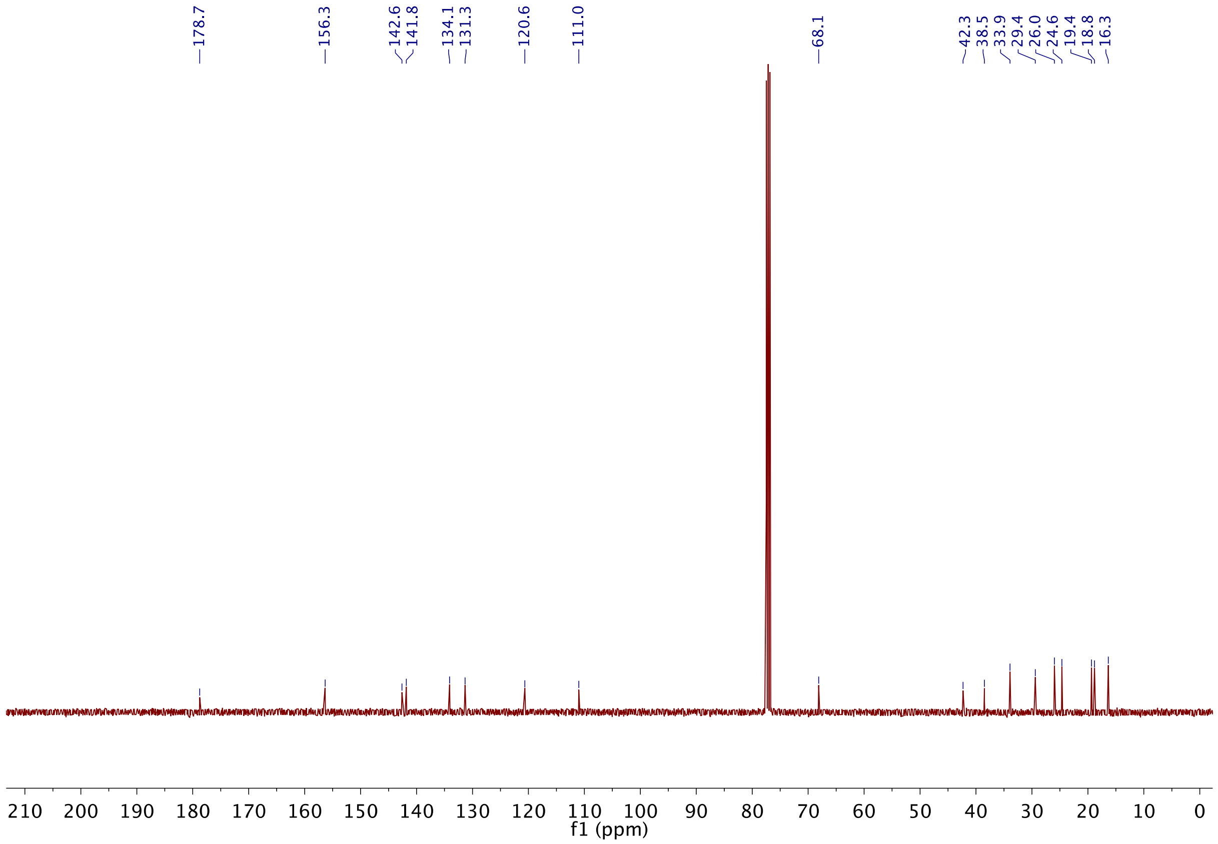

Figure S13. ^{13}C-NMR spectrum of ***E*-1** ($CDCl_3$, 20 °C).

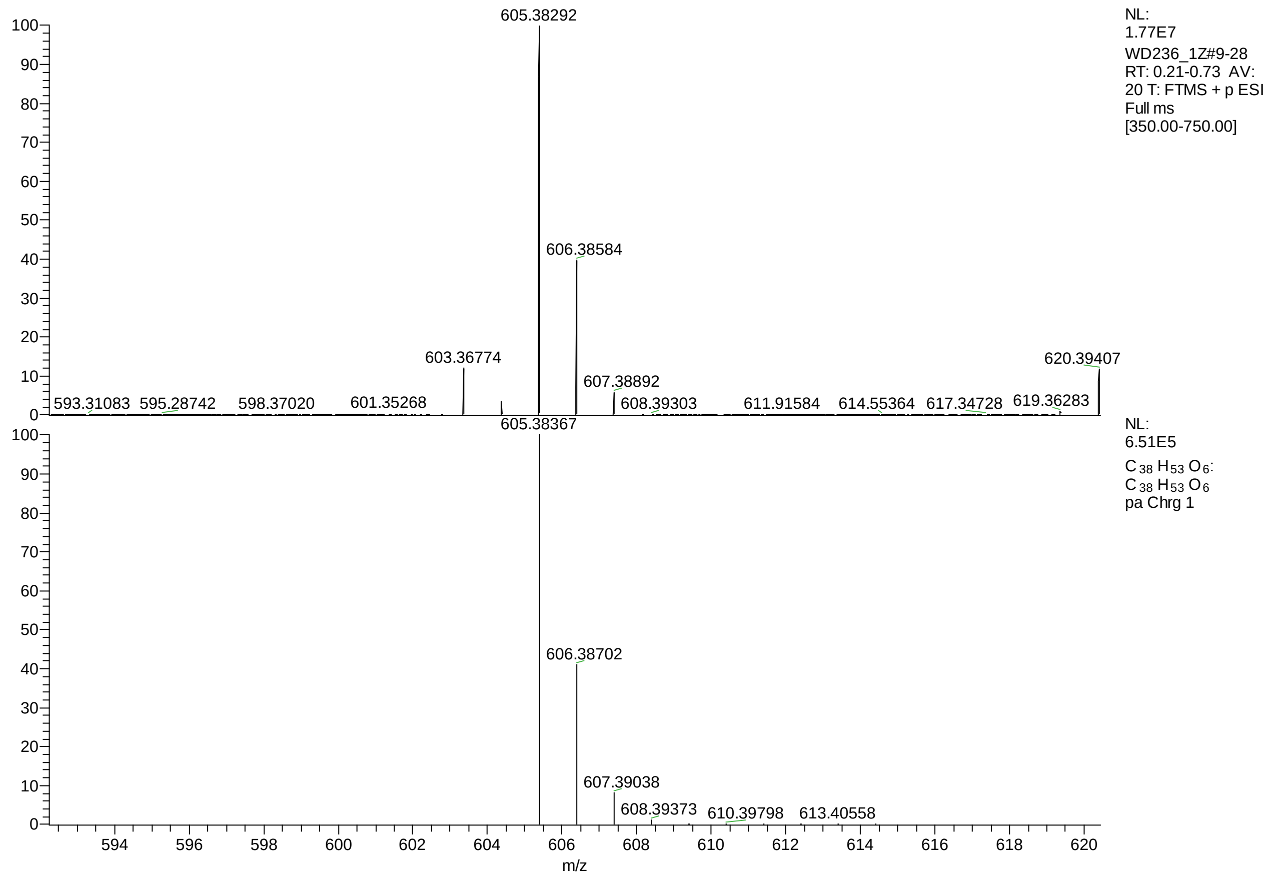

Figure S14. HRMS of ***Z*-2** (top: measured; bottom: calcd).

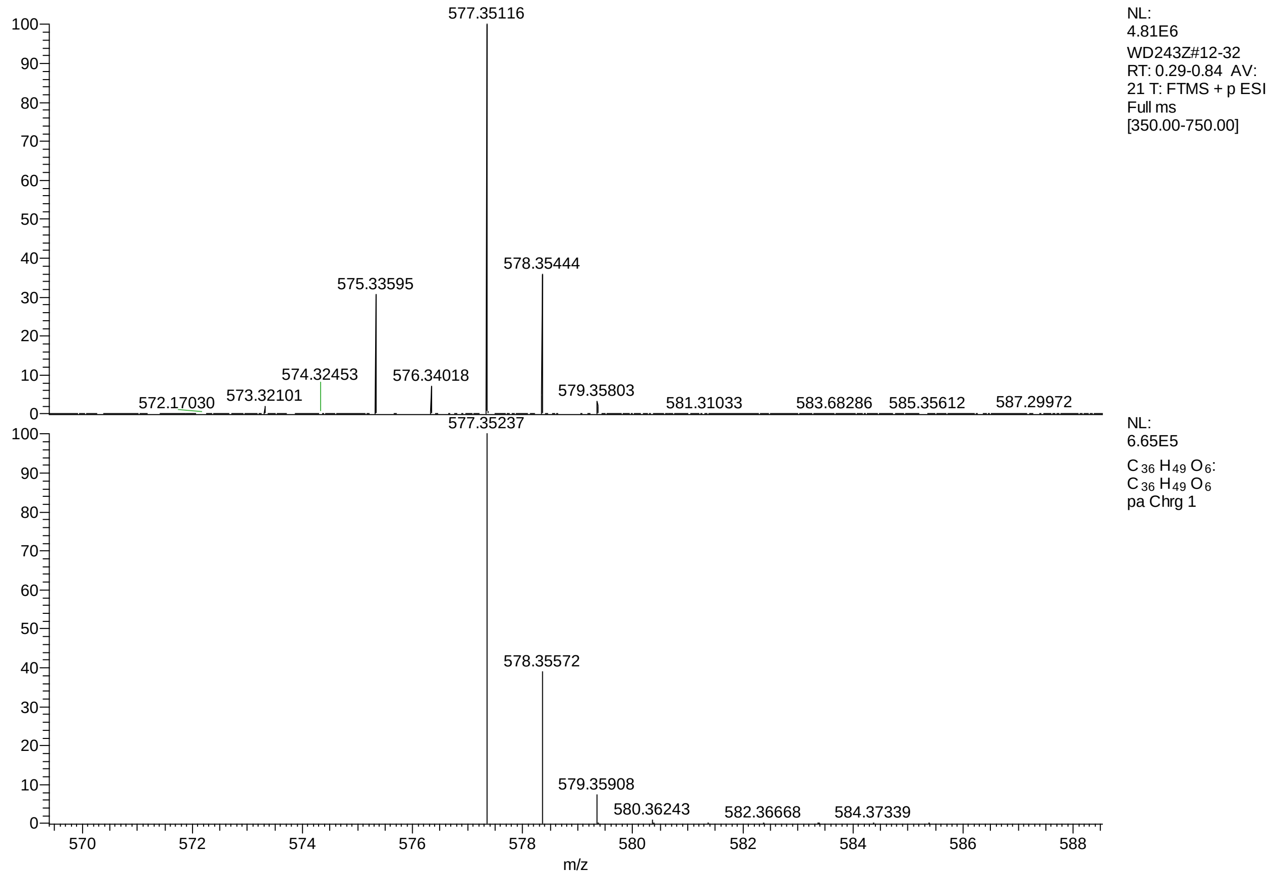

Figure S15. HRMS of ***Z*-1** (top: measured; bottom: calcd).

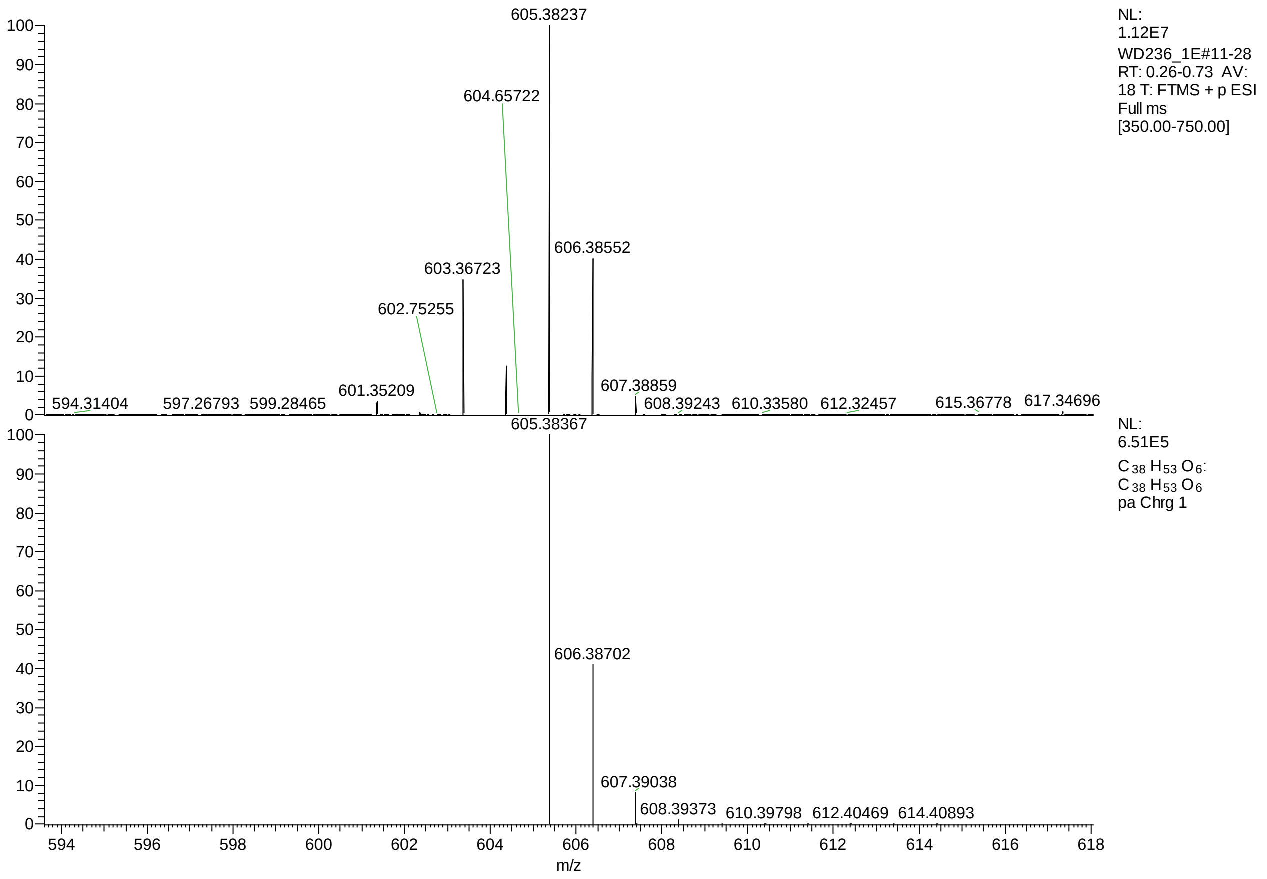

Figure S16. HRMS of ***E*-2** (top: measured; bottom: calcd).

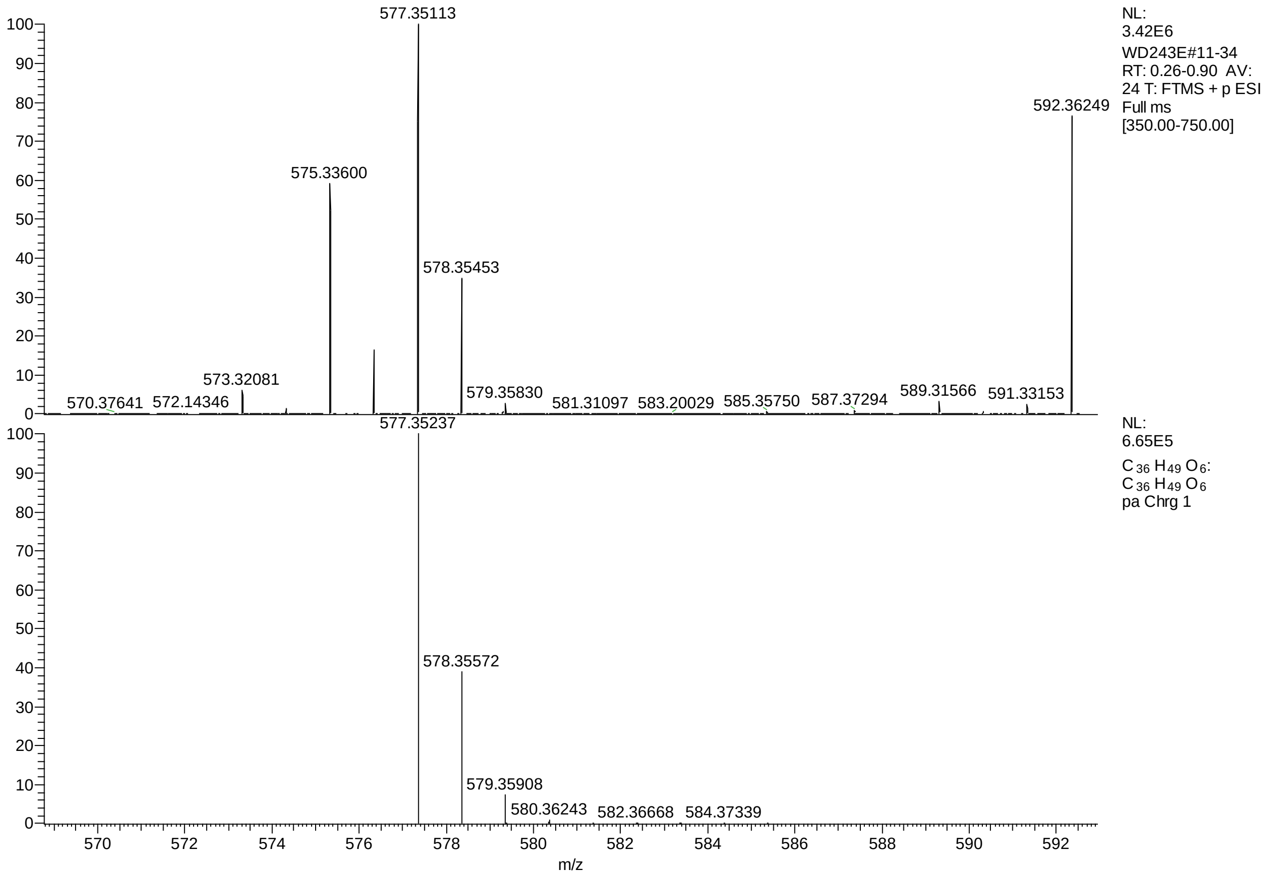

Figure S17. HRMS of ***E*-1** (top: measured; bottom: calcd).

4. Analysis of the rotary motion of molecular motor in solution

The rotary motion of molecular motor *E*/*Z*-**1** was studied in solution using ^{1}H NMR, UV/Vis absorption and CD spectroscopy (Scheme S3a, Figures S18-24). Photochemical isomerization steps *E*-**1**-stable→*Z*-**1**-metastable (Process 1, **Error! Reference source not found.**a, Figure S22) and *Z*-**1**-stable → *E*-**1**-metastable (Process 3, **Error! Reference source not found.**a, Figure S23) were studied with ^{1}H NMR spectroscopy to determine the ratios of the respective metastable and stable isomers at the photostationary state (PSS) established upon irradiation at 312 nm. For *E*-**1**-stable → *Z*-**1**-metastable photoisomerization, a characteristic downfield shift of the resonances assigned to the aliphatic protons were observed in line with the formation of the strained diastereomer. Photoisomerization from *Z*-**1**-stable to *E*-**1**-metastable shifted most of the resonances of the aliphatic protons downfield. Conversely, an upfield shift of the resonance corresponding to the methyl group adjacent to the stereogenic center was observed, characteristic for the *E*-metastable diastereomer of 1st generation molecular motors. Heating of the irradiated samples led to a clean conversion of the metastable isomers to stable isomers, which could be readily identified and characterized with ^{1}H NMR spectroscopy (Figure S22, Figure S23 blue spectra)

Accordingly, for both of these UV-induced processes a large bathochromic shift of the main absorption band was observed in the UV/Vis absorption spectra consistent with the formation of the metastable isomers (Figures Figure S18a and Figure S20a). Furthermore, isosbestic points at 257 nm for *Z*-**1**-stable → *E*-**1**-metastable (Figure S20) and at 263 nm and 330 nm for *E*-**1**-stable → *Z*-**1**-metastable were maintained throughout both photochemical isomerizations (Processes 1 and 3, **Error! Reference source not found.**a) indicating clean unimolecular processes. For both *E*-**1**-stable → *Z*-**1**-metastable and *Z*-**1**-stable → *E*-**1**-metastable processes, CD spectroscopy revealed inversion of the CD signal, corresponding to the lowest-energy transition, upon irradiation at 312 nm (Figure S18b,c and Figure S20b,c). This is in line with the formation of the isomers with opposite helical chirality. The thermal steps (Process 2 and 4, **Error! Reference source not found.**a) were also studied by UV/Vis absorption spectroscopy. Upon thermal helix inversion, the metastable isomers were cleanly converted to their respective stable isomer. During this process, isosbestic points at 251 nm and 233 nm *E*-**1**-metastable→*E*-**1**-stable (Figure S19a) and at 267 nm and 335 nm *Z*-**1**-metastable→*Z*-**1**-stable (Figure S21a) were maintained. CD spectroscopy showed an inversion of signal during photoisomerization, in agreement with the inversion of the helical chirality of the motors upon conversion to the respective metastable isomers (Figure S18b,c Process 1and Figure S20b,c Process 3). Consequently, during the thermal helix inversion steps (Processes 2 and 4, **Error! Reference source not found.**a) another inversion of the CD signal, in line with the recovery of the original helical chirality by the motor (Figure S19b,c and Figure S21b,c). The barriers for the thermal helix inversions (Processes 2 and 4) were determined by Eyring analysis (Figure S19d,c and Figure S21d,c). A value of $\Delta^{‡}G$ (20 °C) = 100.1 ± 0.4 kJ mol^{-1} ($t_{1/2}$ = 21 h) was found for the THI from *Z*-**1**-metastable→*Z*-**1**-stable. As expected, the THI of *E*-**1**-metastable to *E*-**1**-stable was found to be fast at room temperature, $\Delta^{‡}G$ (20 °C) = 78 ± 6 kJ mol^{-1} ($t_{1/2}$ = 10 s).

Finally, irradiation of the sample of stable *Z*-**1** isomer at 312 nm at room temperature led to essentially same photostationary state mixture (~27:73 ratio of stable *E*-**1** to metastable *Z*-**1** isomers as inferred from integration of ^{1}H NMR resonances) as the photostationary state mixture obtained starting from stable *E*-**1** isomer (Figure S22, Figure S24), thus demonstrating that in solution these motors operate as three switches at ambient conditions (**Error! Reference source not found.**b).

In conclusion, these measurements demonstrated that the photochemical and thermal isomerization behavior of this overcrowded-olefin molecular motor scaffold are only marginally affected by the

substitution used in this study. Based on this data, it can thus be concluded that molecular motor *E/Z*-**1** behaves like the structurally related parent diol motor **3**.[1]

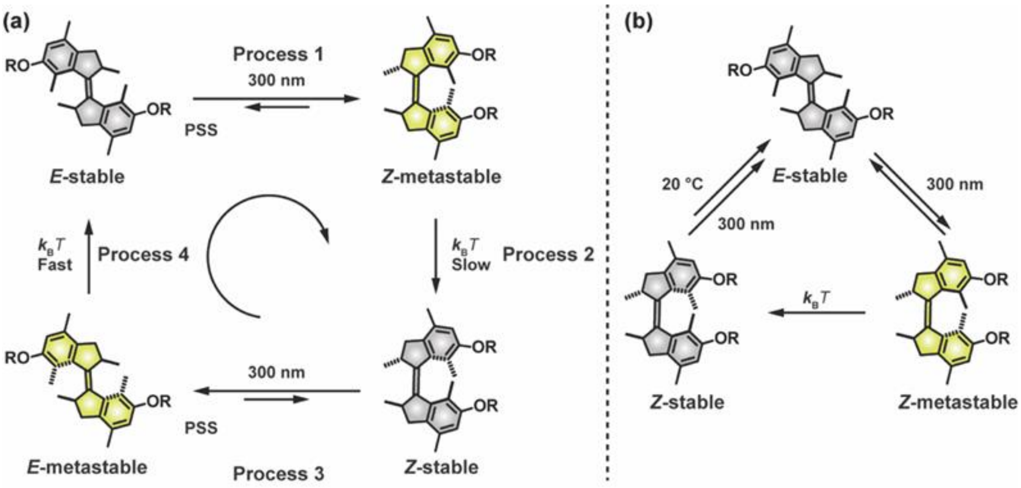

Scheme S3. (a) Light and heat driven unidirectional rotary cycle of molecular motor ***E/Z*-1**. (b) Isomerization pathways and accessible isomers of molecular motor ***E/Z*-1** at room temperature.

4.1 UV/Vis Absorption spectroscopy and CD spectroscopy

Process 1

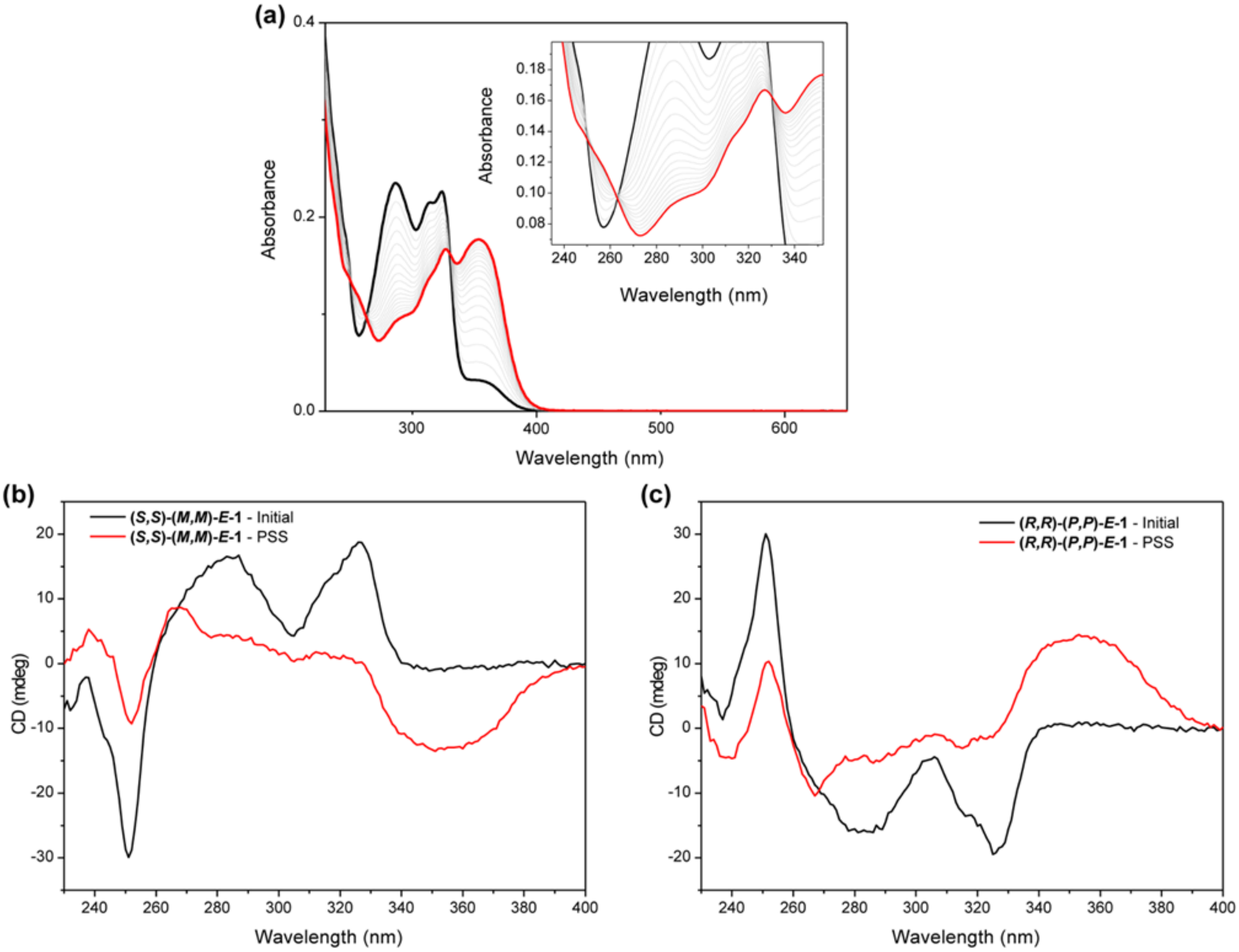

Figure S18. (a) UV/Vis absorption spectrum of *E*-**1** before (black line) and after irradiation to the PSS (red line) at 300 nm (THF). Isosbestic points are maintained at 263 nm and 330 nm. (b) Changes in the CD spectrum of (*S*,*S*)-(*M*,*M*)-*E*-**1** upon irradiation with 312 nm. (c) Changes in the CD spectrum of **(***R*,*R*)-(*P*,*P*)-*E*-**1** upon irradiation with 312 nm.

Process 2

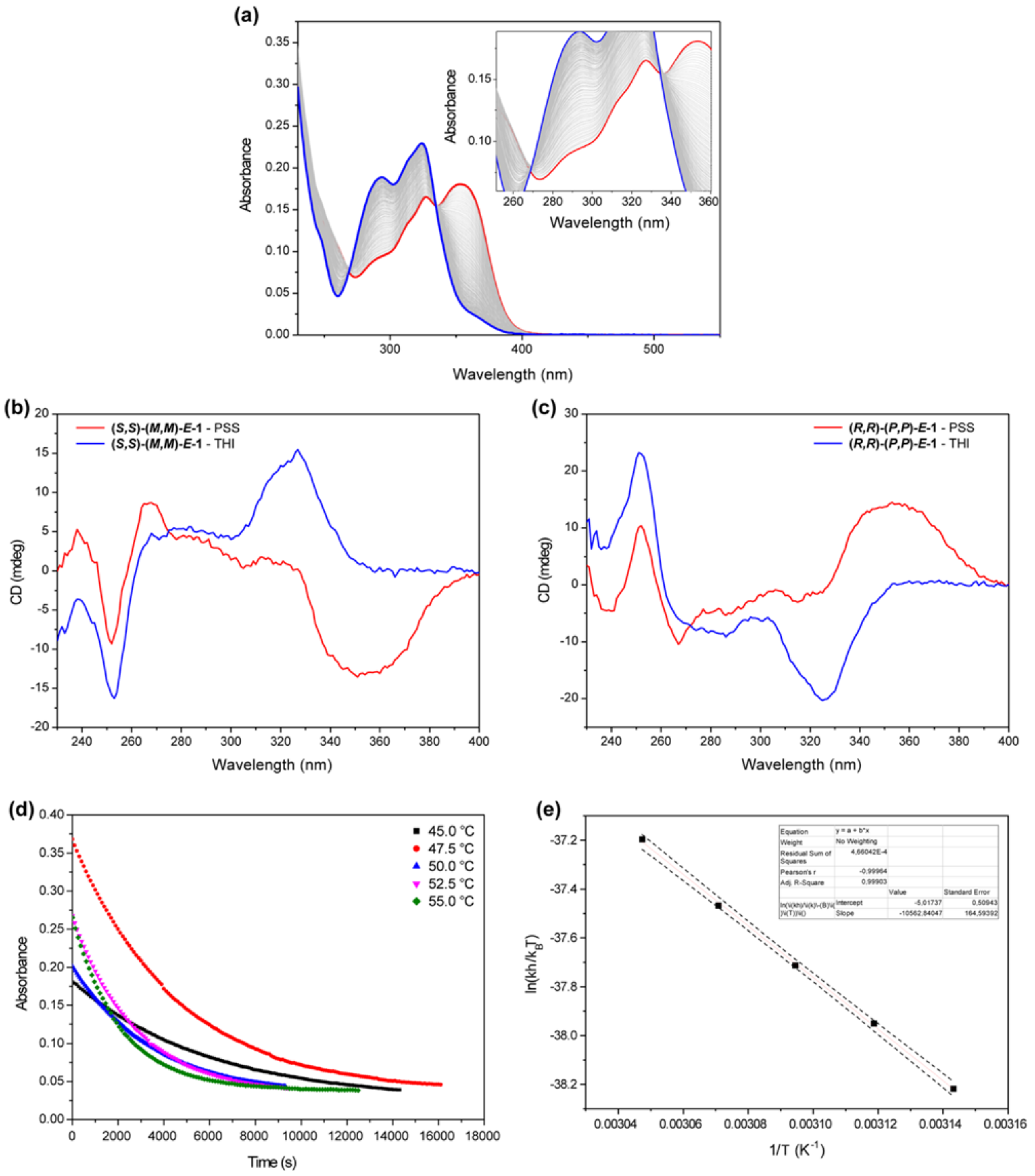

Figure S19. (a) UV/Vis absorption spectrum of the PSS mixture generated from *E*-**1** (red line) and after thermal helix inversion (blue line) (THF). Isosbestic points are maintained at 267 nm and 335 nm. (b) Changes in the CD spectrum of (*S*,*S*)-(*M*,*M*)-*E*-**1** PSS upon thermal helix inversion. (c) Changes in the CD spectrum of **(***R*,*R*)-(*P*,*P*)-*E*-**1** PSS upon thermal helix inversion. (d) Changes in absorption at 355 nm over time during the thermal helix inversion of metastable *Z*-**1** to stable *Z*-**1** at various temperatures (45 – 55 °C every 2.5 °C). (e) Eyring plot analysis of thermal isomerization step from metastable *Z*-**1** to stable *Z*-**1** in THF. Thermodynamic parameters of the transition state ($\Delta^{\ddagger}G$ (20 °C) = 100.1 ± 0.4 kJ mol^{-1}, $t_{1/2}$ = 21 h) were obtained by fitting the linearized form of the Eyring equation using Origin software. Dashed lines indicate 95% confidence intervals.

Process 3

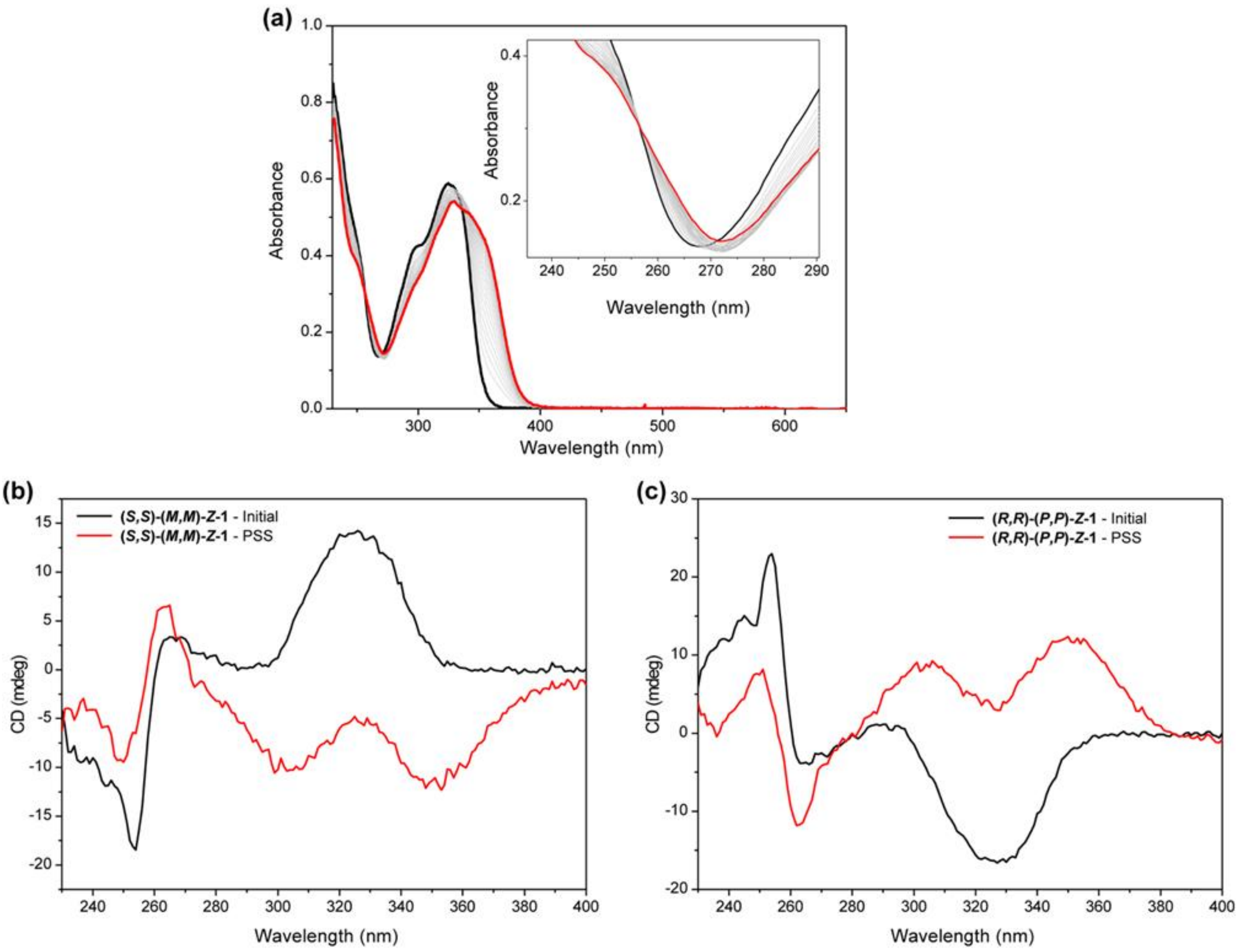

Figure S20. (a) UV/Vis absorption spectrum of *Z*-**1** before (black line) and after irradiation to the PSS (red line) at 300 nm (THF, at -20 °C). An isosbestic point is maintained at 257 nm. (b) Changes in the CD spectrum of (*S*,*S*)-(*M*,*M*)-*Z*-**1** upon irradiation with 312 nm. (c) Changes in the CD spectrum of **(***R*,*R*)-(*P*,*P*)-*Z*-**1** upon irradiation with 312 nm.

Process 4

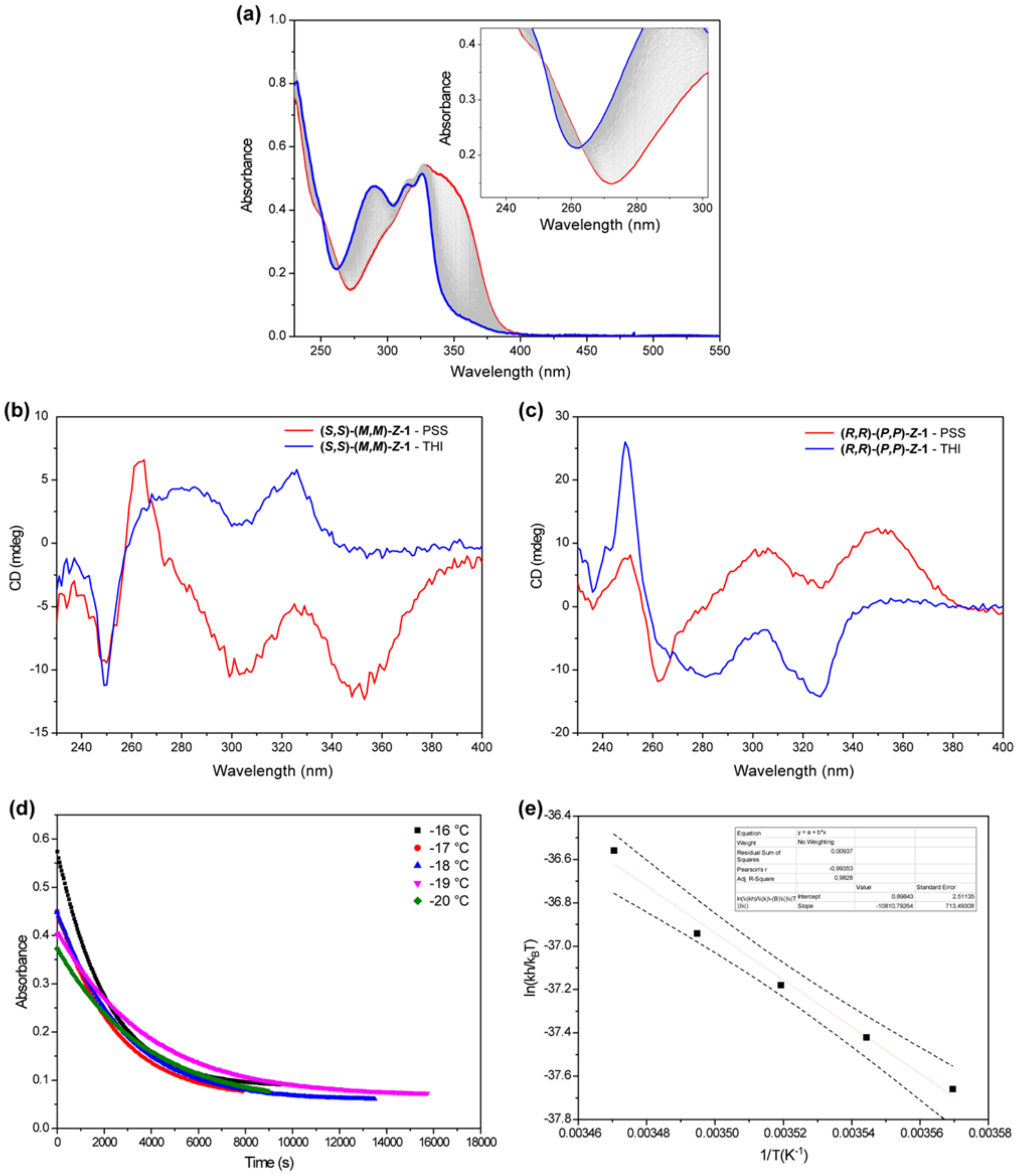

Figure S21. (a) UV/Vis absorption spectrum of the PSS mixture generated from *Z*-**1** (red line) and after thermal helix inversion (blue line). Isosbestic points are maintained at 233 nm and 251 nm. (b) Changes in the CD spectrum of (*S*,*S*)-(*M*,*M*)-*Z*-**1** PSS upon thermal helix inversion. (c) Changes in the CD spectrum of **(***R*,*R*)-(*P*,*P*)-*Z*-**1** PSS upon thermal helix inversion. (d) Changes in absorption at 355 nm over time during the thermal helix inversion of metastable *E*-**1** to stable *E*-**1** at various temperatures (-16 – 20 °C every 1.0 °C). (e) Eyring plot analysis of thermal isomerization step from metastable *Z*-**1** to stable *Z*-**1** in THF. Thermodynamic parameters of the transition state ($\Delta^{\ddagger}G$ (20 °C) = 78 ± 6 kJ mol^{-1}, $t_{1/2}$ = 10 s) were obtained by fitting the linearized form of the Eyring equation using Origin software. Dashed lines indicate 95% confidence intervals.

4.2 ^{1}H NMR studies

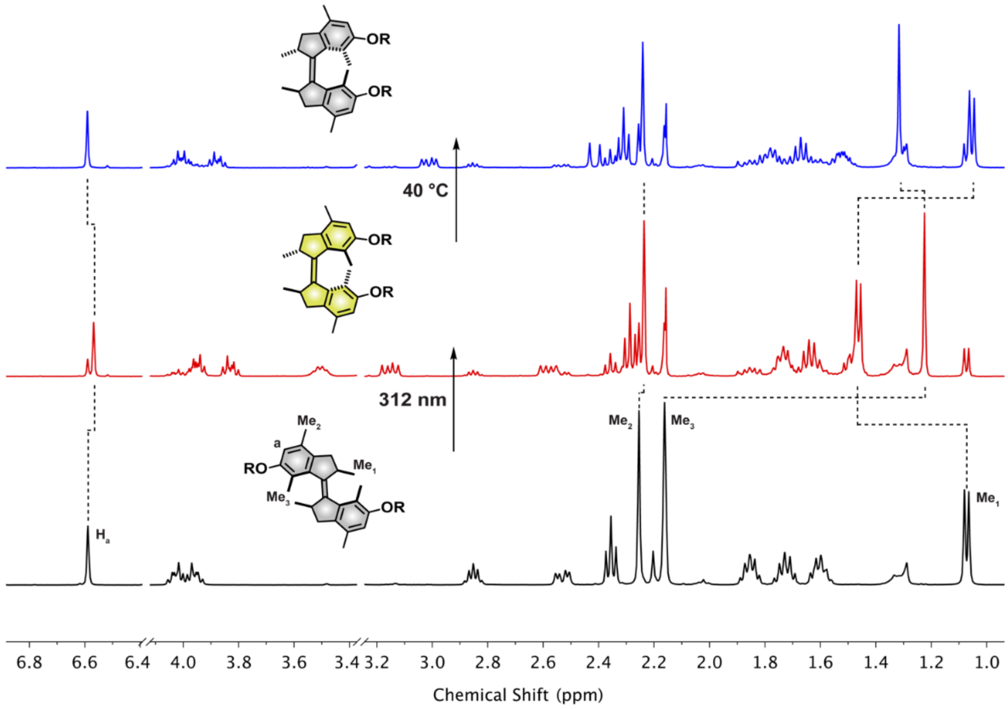

Figure S22. ^{1}H-NMR spectra of *E*-**1** before (black spectrum), upon irradiation with 312 nm light at -5 °C to the PSS, PSS = 75:25 (determined by the integration of resonances corresponding to a and Me_1 protons) of metastable *Z*-**1** to stable *E*-**1** (red spectrum), and after subsequent heating (40 °C, overnight) to induce THI of metastable *Z*-**1** stable *Z*-**1** (blue spectrum). All spectra recorded in methanol-d_4 at RT.

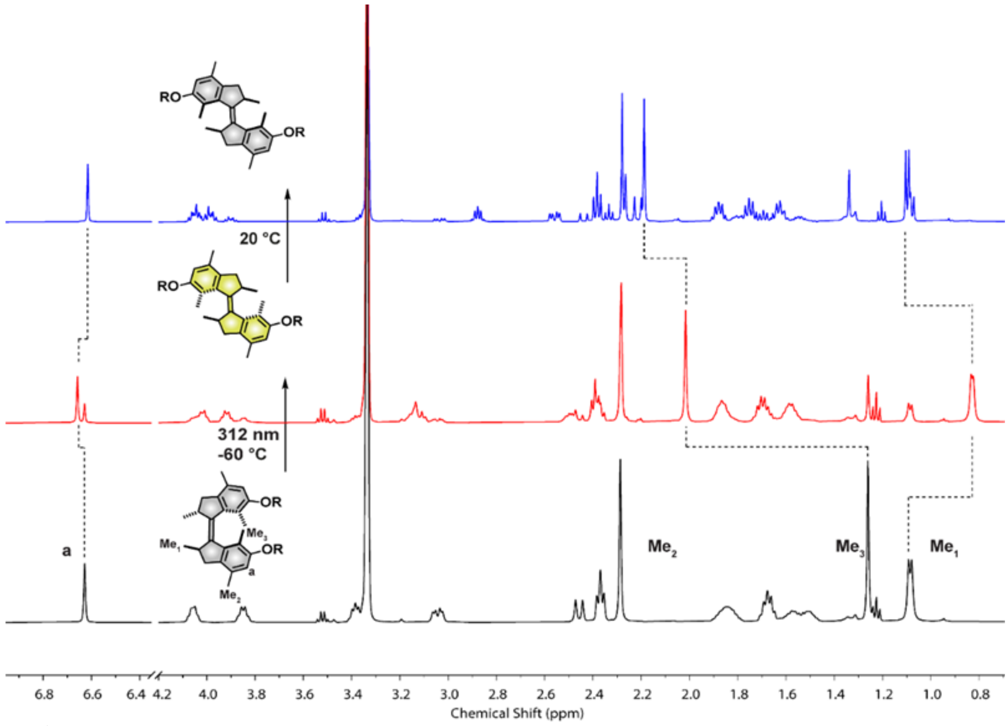

Figure S23. ^{1}H-NMR spectra of *Z*-**1** before (black spectrum), photostationary state mixture of metastable *E*-**1** and stable *Z*-**1,** PSS ratio of metastable *E*-**1**:stable *Z*-**1,** 71:29 (determined by the integration of resonances corresponding to a and Me_1 protons), generated upon irradiation with 312 nm at -60 °C (red spectrum), and after THI of metastable *E*-**1** stable *E*-**1** (blue spectrum). All spectra recorded in methanol-d_4 at -60 °C. Resonances corresponding to ethanol originate from the cooling bath solvent adsorbed on the outside of the NMR tube.

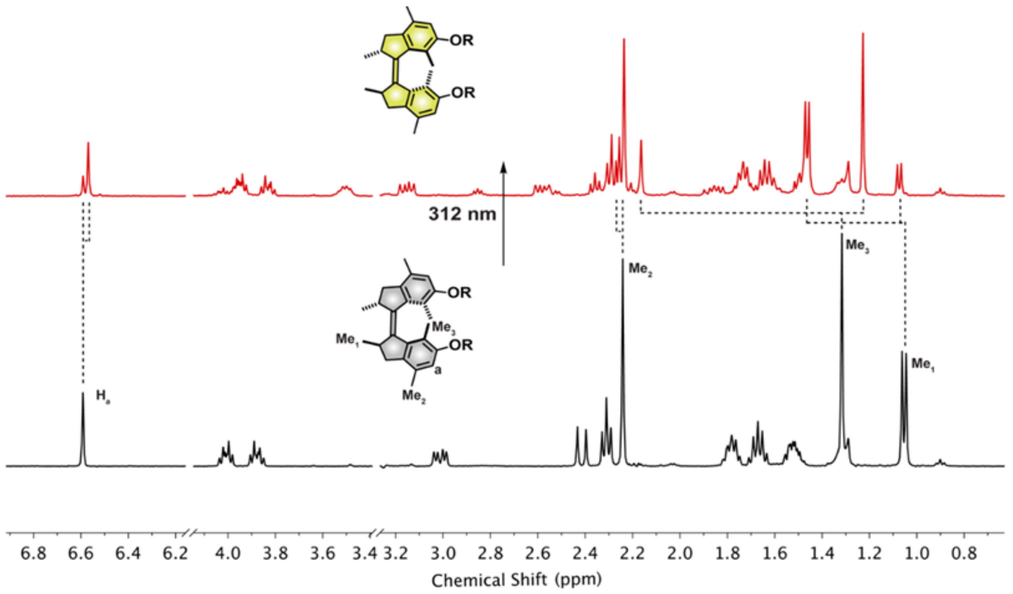

Figure S24. ^{1}H-NMR spectraa of *Z*-**1** before (black spectrum), photostationary state mixture of metastable
Z-**1** and stable *E*-**1,** PSS ratio = metastable *Z*-**1**:stable *E*-**1** = 73:27, generated upon irradiation with 312 nm at room temperature (red spectrum). All spectra recorded in methanol-d_4 at 20 °C.

4.3 Raman Studies in solution

In addition, isomerization cycle of motor **1** was followed in solution by Raman spectroscopy. It was found that the respective diastereoisomers could be readily identified by Raman shifts of the characteristic bands. Irradiation of stable *E*-**1** solution at 300 nm to gradual decrease in intensity centered at 1626 cm^{-1} ascribed to the aromatic ring mode of the stable isomer and gradual appearance of a new band centered at 1565 cm^{-1}, in line with the formation of the metastable *Z*-**1** (Figure S25a). Heating of this sample resulted in a gradual decrease in the intensity of the band centered at 1565 cm^{-1} accompanied by the increase in the intensity of the band at 1636 cm^{-1} (Figure S25b). The shift of the band characteristic of the stable isomer from 1626 cm^{-1} to 1636 cm^{-1} is consistent with formation of the stable *Z*-**1** isomer from the metastable isomer and demonstrates that these species can be clearly distinguished with Raman spectroscopy (Figure S25c). Consequently, irradiation at 300 nm of the solution of stable *Z*-**1** at -50 ºC led to gradual decrease in the intensity of the band centered 1638 cm^{-1} and appearance of the new band at 1573 cm^{-1} (Figure S26a) while heating of this sample resulted in the formation of new band at 1629 cm^{-1} (Figure S26b) consistent with the sequential stable *Z*-**1** to metastable *E*-**1** to stable *E*-**1** isomerization photochemical and thermal isomerization sequence (Figure S26c). Finally, irradiation of the stable *Z*-**1** solution at RT resulted in a gradual decrease of the band characteristic of stable *Z*-**1** (1638 cm^{-1}) with concomitant appearance of the band characteristic of the stable *E*-**1** isomer (1626 cm^{-1}) and finally formation of the band characteristic of metastable *Z*-**1** isomer (1565 cm^{-1}) in line with the formation of the metastable *Z*-**1** via unidirectional sequence of photochemical and thermal isomerization reactions typical of 1st generation molecular motors (Figure S27d, **Error! Reference source not found.**a,b).

Process 1 and Process 2

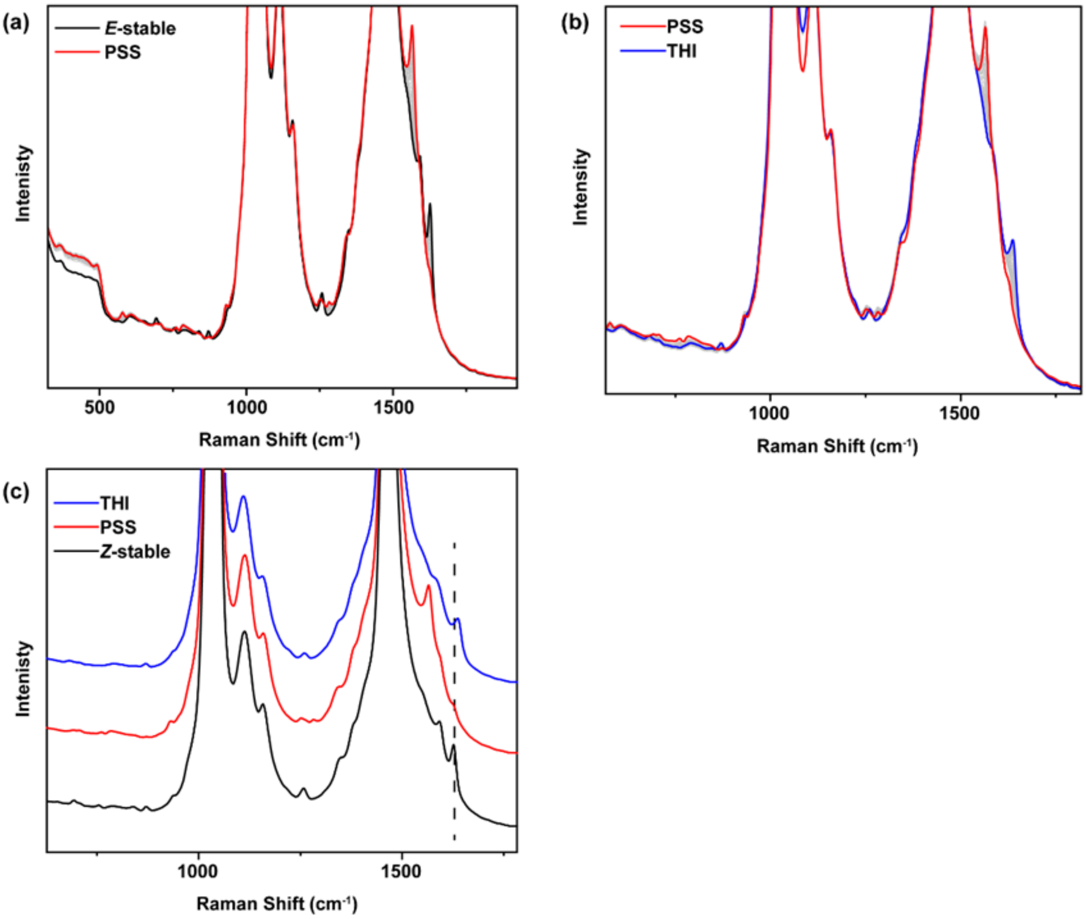

Figure S25. (a) Changes in the Raman spectrum (785 nm, 400 mW, MeOH, RT) of stable *E*-**1** (black line) during irradiation at 300 nm until the photostationary state (PSS, red line). (b) Changes in the Raman spectrum of the PSS mixture generated from *E*-**1** (red line) during thermal helix inversion at 60 ºC until full conversion of the metastable *Z*-**1** to stable *Z*-**1** isomer (blue line). Grey lines indicate intermediate spectra. (c) Comparison of Raman spectra of stable *E*-**1** (black line), PSS mixture (red line) and after thermal helix inversion (blue line). Dashed line is guide for the eyes highlighting characteristic bands.

Process 3 and Process 4

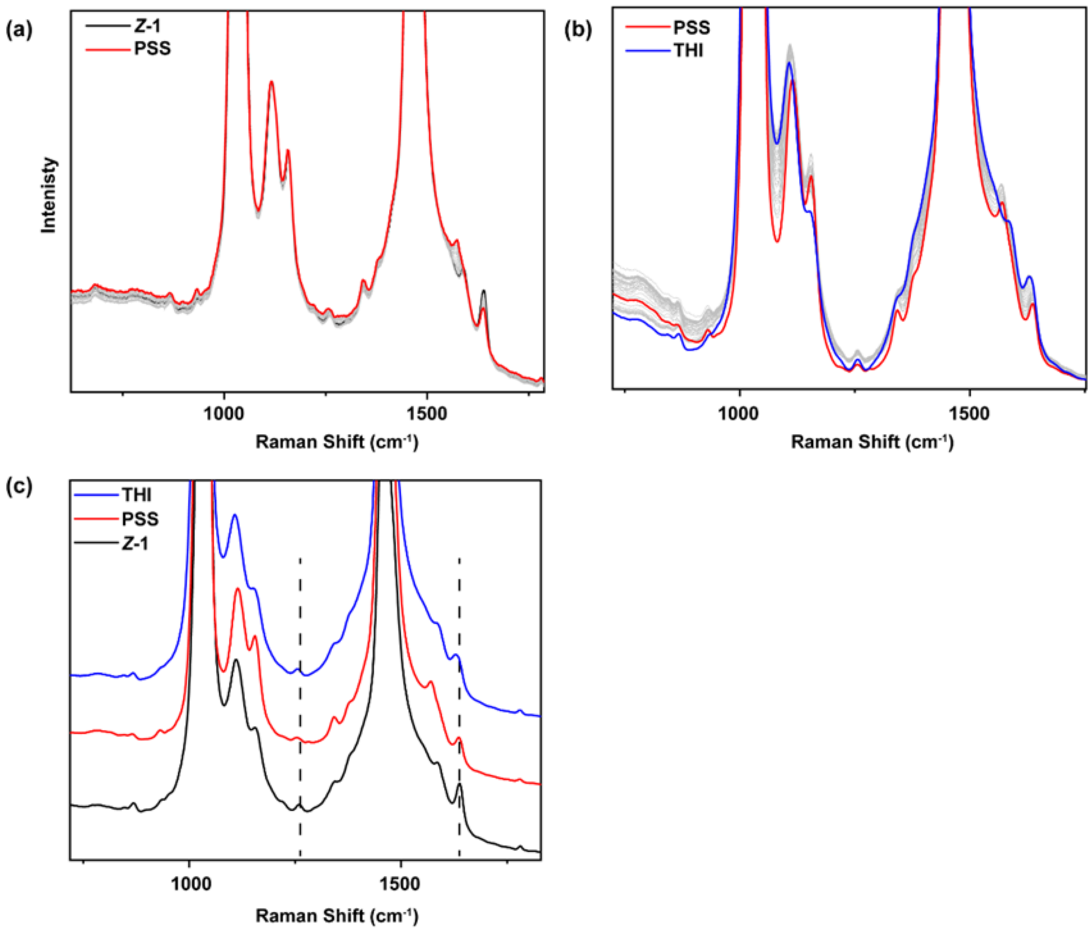

Figure S26. (a) Changes in the Raman spectrum (785 nm, 400 mW, MeOH, -50 ºC) of stable *Z*-**1** (black line) during irradiation at 300 nm until the photostationary state (PSS, red line). (b) Changes in the Raman spectrum of the PSS mixture generated from *Z*-**1** (red line) during thermal helix inversion upon warming of the sample to RT until full conversion of the metastable *E*-**1** to stable *E*-**1** isomer (blue line). Grey lines indicate intermediate spectra. Inconsistent changes in the background scattering originate from ice thawing on the outside of the sample holder. (c) Comparison of Raman spectra of stable *E*-**1** (black line), PSS mixture (red line) and after thermal helix inversion (blue line). Dashed lines are guide for the eyes highlighting characteristic bands.

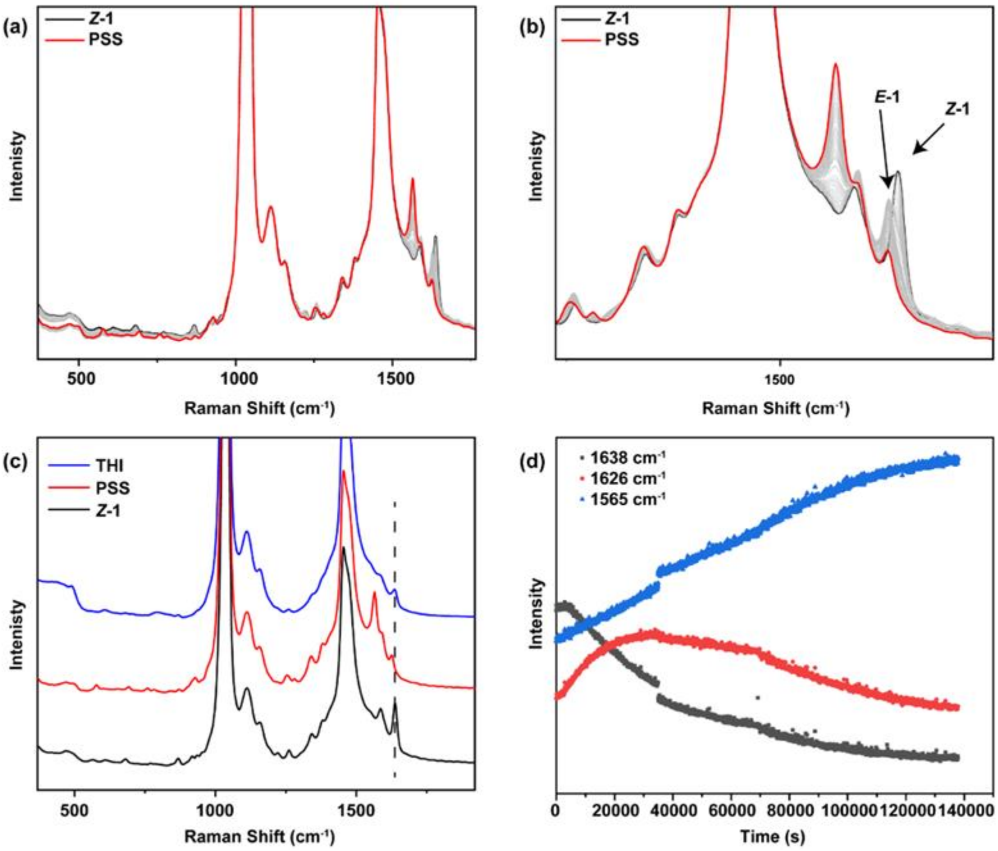

Figure S27. (a) Changes in the Raman spectrum (785 nm, 400 mW, MeOH, RT) of stable *Z*-**1** (black line) during irradiation at 300 nm until the photostationary state (PSS, red line). (b) Expansion of the region with characteristic of stable *Z*-**1** and *E*-**1** isomers. The characteristic bands were marked with the arrows. Grey lines indicate intermediate spectra. Inconsistent changes in the background scattering originate from ice thawing on the outside of the sample holder. (c) Comparison of Raman spectra of stable *Z*-**1** (black line), PSS mixture (red line) and after thermal helix inversion (blue line). Dashed lines are guide for the eyes highlighting characteristic bands. (d) Changes in the intensity of the characteristic bands in the Raman spectrum upon irradiation of stable *Z*-**1** at 300 nm at RT

5. Raman studies in the solid state

Raman micropectroscopy was proved to be a suitable tool to follow the photochemical and thermal isomerization of molecular motors *E*/*Z*-**1** in the solid state. In line with the studies performed in the solution, both powdered samples of *E*/*Z*-**1** showed significant differences in the Raman shifts of the bands that were ascribed to aromatic ring mode and overcrowded double bond stretching (Figure S28a,b). In addition, several other characteristic bands were found in the fingerprint region of Raman spectra of both *E*/*Z*-**1** motors, allowing for unambiguous identification of either of the diasteroisomers. Irradiation of the drop casted sample of *Z*-**1** (Figure S29a) at 300 nm at RT led to the appearance of a new band in the Raman spectrum centered at 1572 cm^{-1} (Figure S30a,b) in line with the formation of the metastable isomer of the molecular motor **1**. Comparison to the data collected in the solution (1573 cm^{-1} and 1565 cm^{-1} for metastable *E*-**1** and *Z*-**1**, respectively) allowed to ascribe this band to the metastable *E*-**1** isomer. Furthermore, the intensity of this band gradually decreased (Figure S31a) over time, in line with the thermal helix inversion step, ultimately yielding a spectrum consistent with stable *E*-**1** isomer (Figure S30a,b,c), thus further supporting formation of the metastable *E*-**1** isomer. The half-life of the metastable *E*-**1** was

calculated by following the gradual decrease in the intensity of the band centered at 1571 cm^{-1} and was found to be remarkably long (ca. $t_{1/2}$ = 50 min.) in comparison to the solution ($t_{1/2}$ = 10 s) even under local-heating caused by the direct exposure to 785 nm 500 mW Raman laser (Figure S31b). It should be noted that the half-life of metastable *E*-**1** in solid is expected to be significantly longer for the sample kept in the dark (without exposure the 785 nm laser). Accordingly, irradiation of the drop casted *E*-**1** sample (Figure S29b) at 300 nm led to appearance of a new band in the Raman spectrum centered at 1562 cm^{-1} (Figure S32a,b) characteristic of the metastable *Z*-**1** (in solution 1573 cm^{-1} and 1565 cm^{-1} for metastable *E*-**1** and *Z*-**1**, respectively). However, in contrast to *E*-**1**, exposure of *Z*-**1** to 300 nm light led to decomposition of the sample which was manifested as a large decrease in the overall scattering intensity in the Raman spectrum upon. Therefore, in order to avoid the complete decomposition, samples were irradiated for 10 min. and consequently the conversion to the metastable *Z*-**1** was low (Figure S32a,b). Furthermore, attempts to mitigate this decomposition by exclusion of oxygen, that is preparation of the sample in a glovebox and subsequent transport and measurement in a sealed quartz cuvette failed, thus indicating that the most probable decomposition pathway is photopolymerization of either stable *E*-**1** or metastable *Z*-**1** under 300 nm UV light. It should be emphasized however, that the Raman studies required much larger sampling area in comparison to the mc-AFM and therefore it can be expected that for the samples used in the mc-AFM measurements the photoconversion of *E*-**1** to the metastable *Z*-**1** is much higher. Half-life of the metastable *Z*-**1** in the solid was calculated by following the gradual decrease in the intensity of the band centered at 1562 cm^{-1} and was found (ca. $t_{1/2}$ = 80 min.) to be similar to the half-life of the metastable *E*-**1** in the solid state (Figure S33a,b). Even though identification of the product of this thermal process was difficult due to the decomposition and low conversion to the metastable *Z*-**1**, appearance of some additional characteristic bands in the Raman spectrum (Figure S32b,c) suggested formation of the stable *Z*-**1** and thus competition of the rotary cycle.

In conclusion, in a stark contrast to the solution, molecular motor **1** in the solid operates as a four-state chiroptical switch at ambient temperatures. Furthermore, in the solid, all four diastereoisomers are readily accessible in a sequential manner upon UV-light and heat treatment and thermal stabilities of both metastable *E*-**1** and *Z*-**1** are similar. Finally, it should be noted that even upon prolonged (>40 min.) irradiation of the identically prepared samples on quartz substrates no signs of photoisomerization or photodecomposition were observed, indicating crucial impact of the Au substrates on the photoisomerization process, most probably by facilitating melting of the samples upon local heating of the substrate.

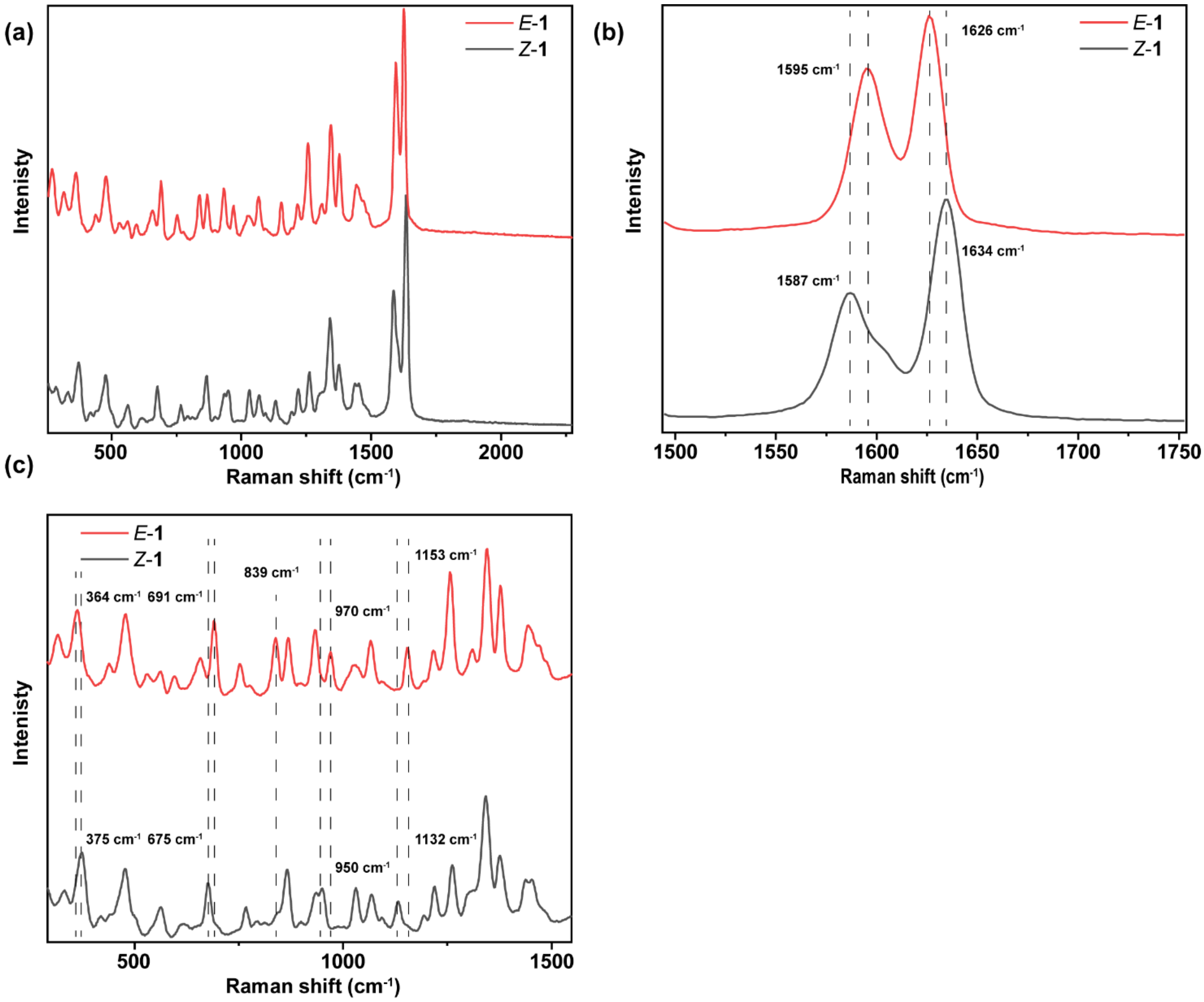

Figure S28. (a) Comparison of Raman spectra (785 nm, 500 mW, Au@Mica) of powdered *Z*-**1** (black line) and *E*-**1** (red line). (b) Expansion of the region with bands ascribed to aromatic ring mode and stretching of the overcrowded double bond. (c) Expansion of the fingerprint region.

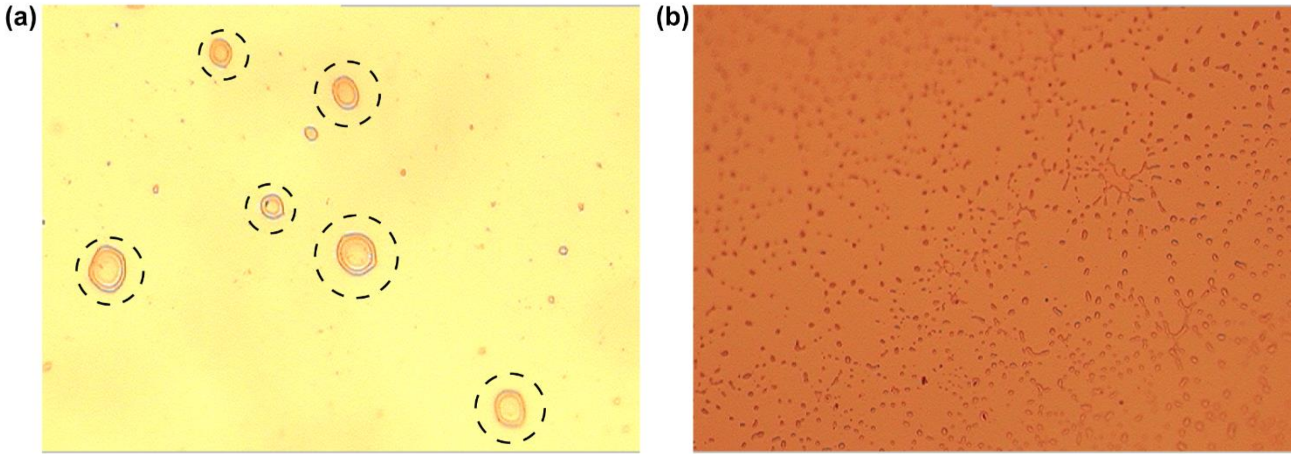

Figure S29. Optical micrographs (100x magnification) of the drop casted (0.1 mg/mL, THF) samples of *Z*-**1** (a) and *E*-**1** (b)

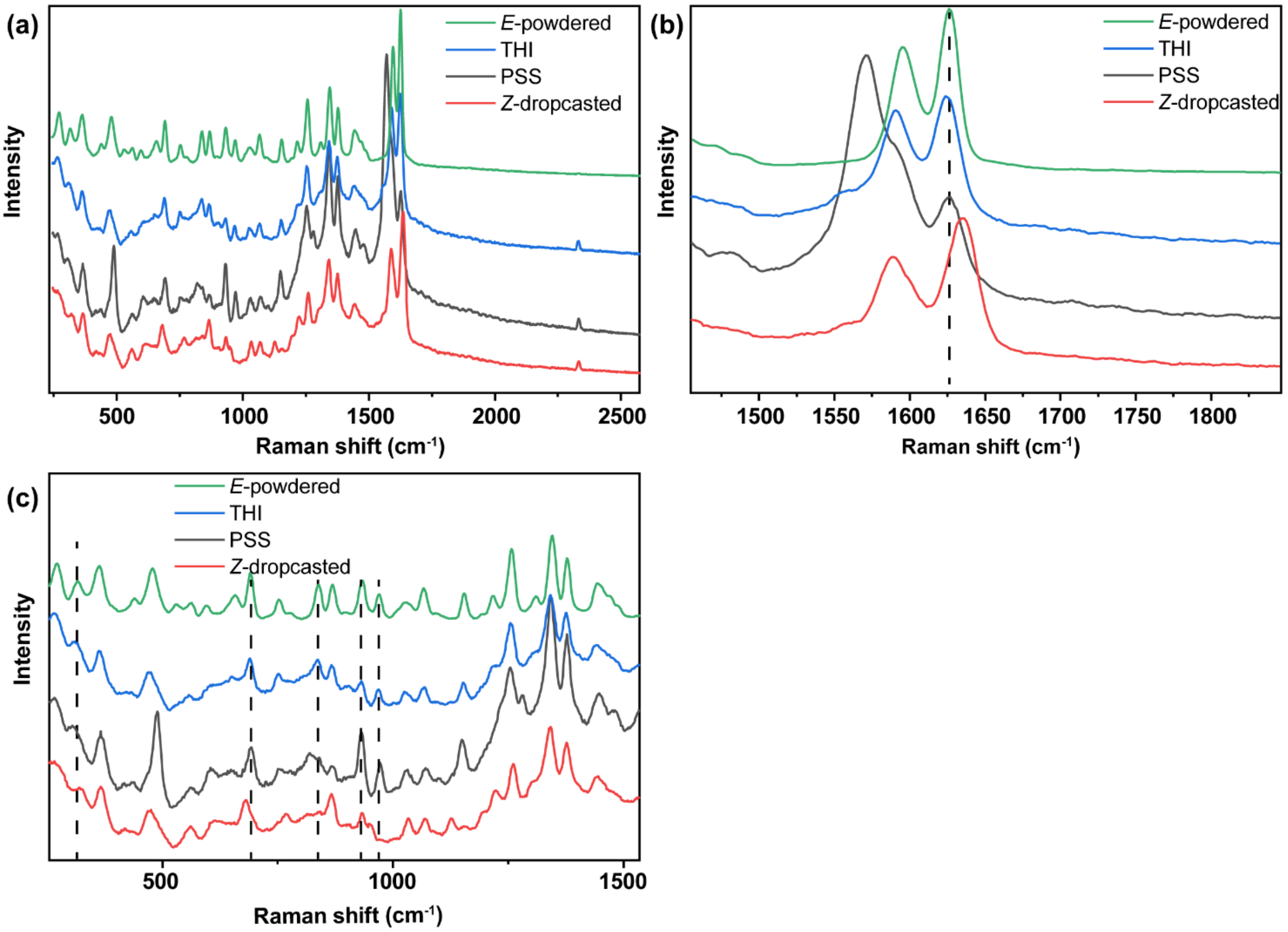

Figure S30. (a) Changes in the Raman spectrum (785 nm, 500 mW, Au@Mica) of the drop casted stable *Z*-**1** (red line) upon irradiation at 300 nm (black line) and subsequent thermal helix inversion (blue line). Raman spectrum of powdered *E*-**1** was added for comparison (green line). (b), (c) Expansion of the spectra in the regions with characteristic bands. Dashed lines are guide for the eyes highlighting characteristic bands.

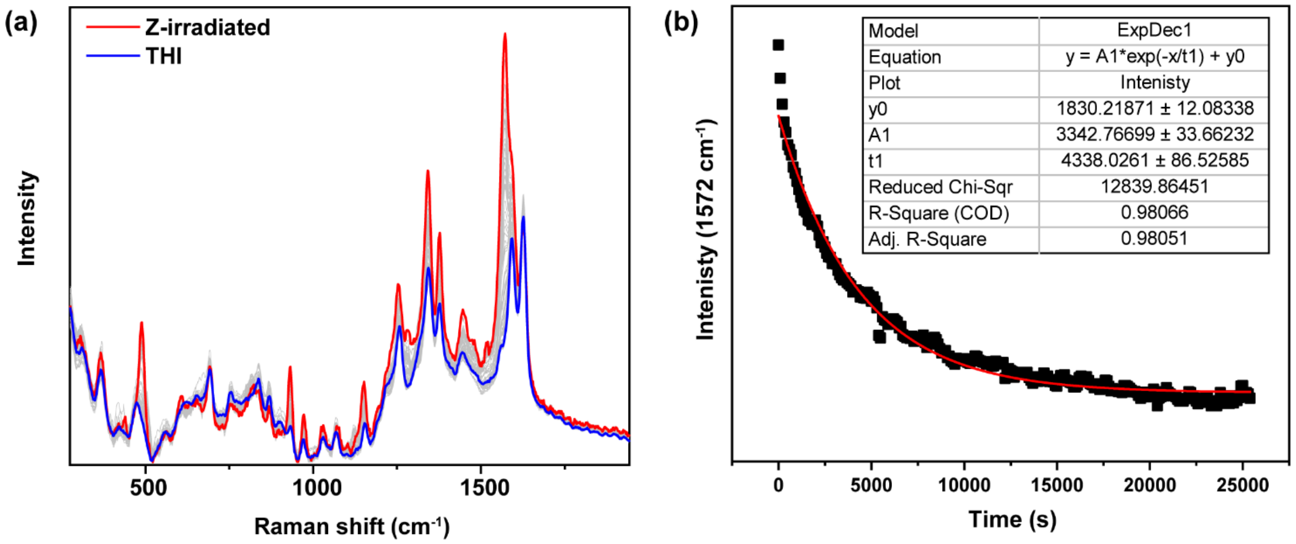

Figure S31. (a) Changes in the Raman spectrum (785 nm, 500 mW, Au@Mica) of the stable *Z*-**1** irradiated at 300 nm (*Z*-irradiated, red line) during thermal helix inversion (THI, blue line). Grey lines indicate intermediate spectra. (b) Changes in the intensity of the band cantered at 1572 cm^{-1} followed in time. Red line indicates least-square fit. Fitting parameters are given in the table.

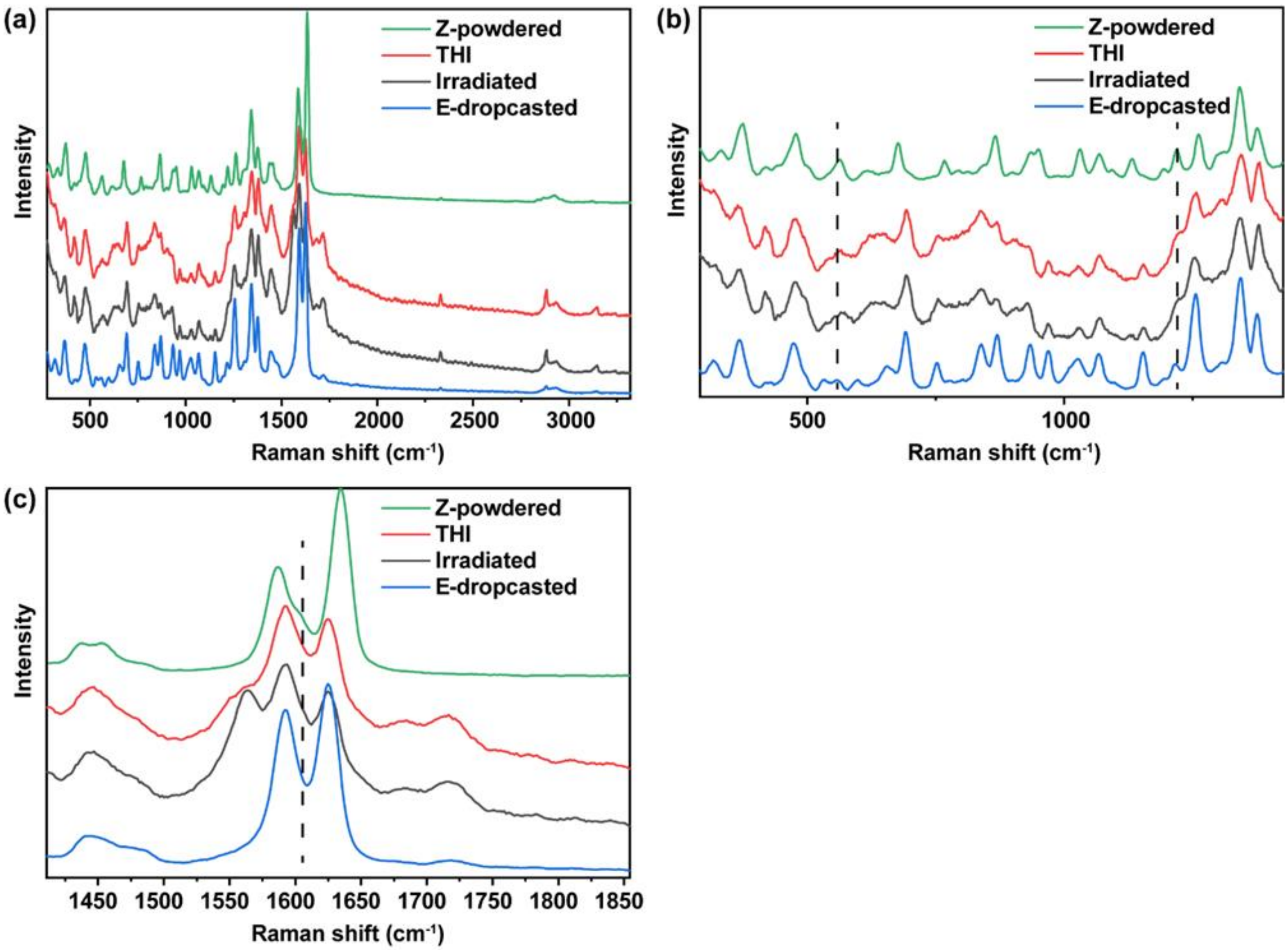

Figure S32. (a) Changes in the Raman spectrum (785 nm, 500 mW, Au@Mica) of the drop casted stable *E*-**1** (red line) upon irradiation at 300 nm (black line) and subsequent thermal helix inversion (blue line). Raman spectrum of powdered *Z*-**1** was added for comparison (green line). (b), (c) Expansion of the spectra in the regions with characteristic bands. Dashed lines are guide for the eyes highlighting characteristic bands.

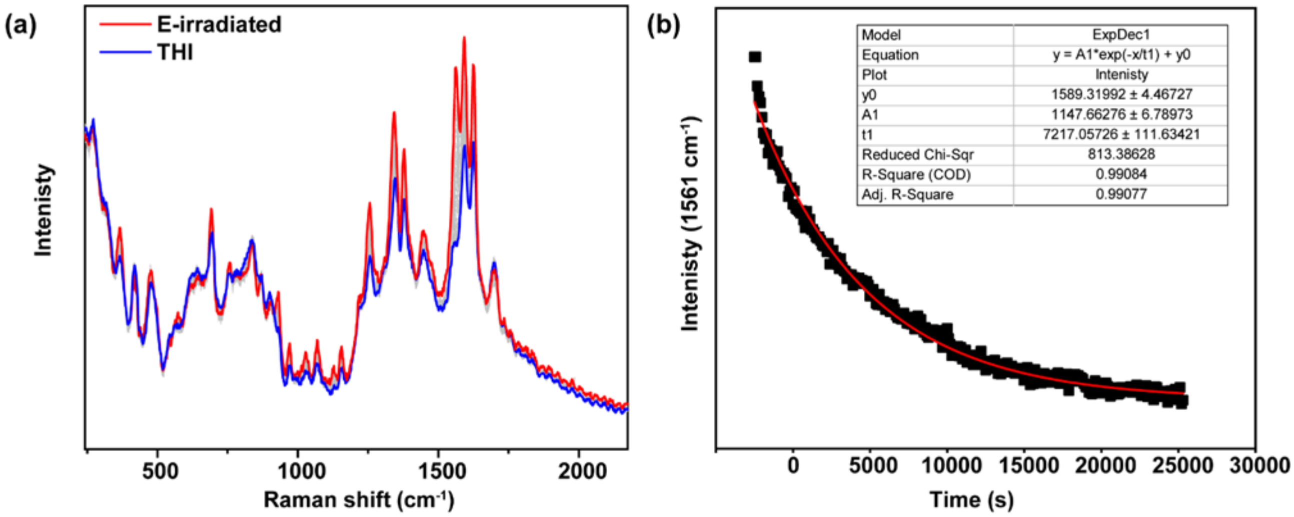

Figure S33. (a) Changes in the Raman spectrum (785 nm, 500 mW, Au@Mica) of the stable *E*-**1** irradiated at 300 nm (*Z*-irradiated, red line) during thermal helix inversion (THI, blue line). Grey lines indicate intermediate spectra. (b) Changes in the intensity of the band cantered at 1561 cm^{-1} followed in time. Red line indicates least-square fit. Fitting parameters are given in the table.

6. CISS measurements in the solid state

Spin-dependent I-V curves were collected from each state of *E* and *Z* molecular motors by mc-AFM in ambient atmosphere. To determine the influence of the CISS effect on the electronic energy levels, the density of states as a function of bias voltage is required. This function is equivalent to dI/dV vs. V curves derived by analytical differentiation of the experimental I-V curves. The gap between the turning points of the resultant U-shaped curves gives the tunneling barrier. The energy gap was determined for the different configurations and field directions. A lower effective barrier means favorable spin selectivity represented by higher spin currents.

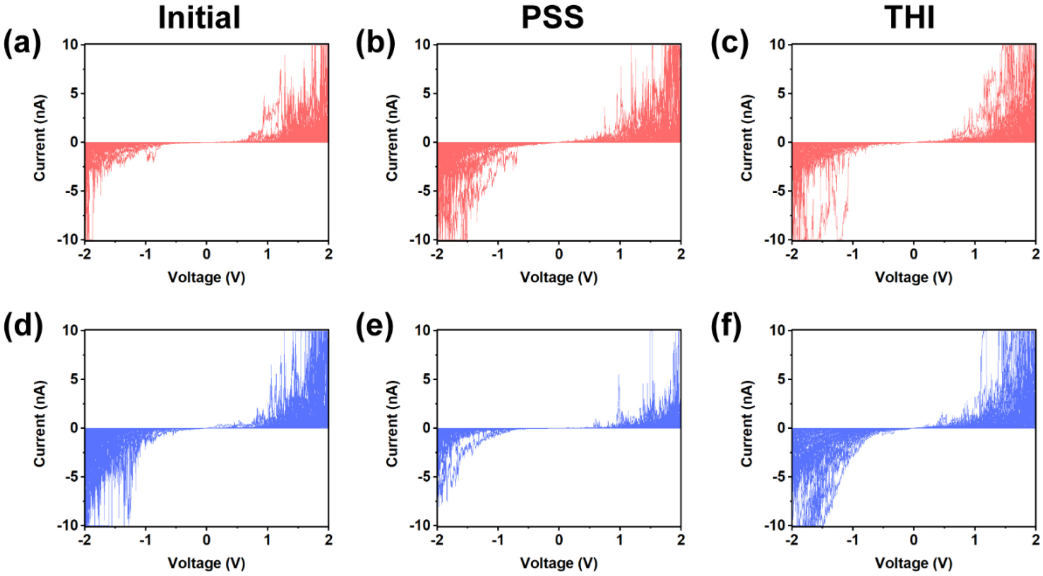

Figure S34. Current versus voltage (I-V) curves (raw data) for the (*S*,*S*)-(*M*,*M*)-*E* layer absorbed on the Ti (10 nm)/Ni (120 nm)/Au (8 nm) substrate with the magnet pointing down (blue) or up (red). (a) and (d) present initial (*S*,*S*)-(*M*,*M*)-*E*. (b) and (e) present irradiated state (*S*,*S*)-(*P*,*P*)-*Z* (PSS). (c) and (f) present thermally relaxed (*S*,*S*)-(*M*,*M*)-*Z* (THI).

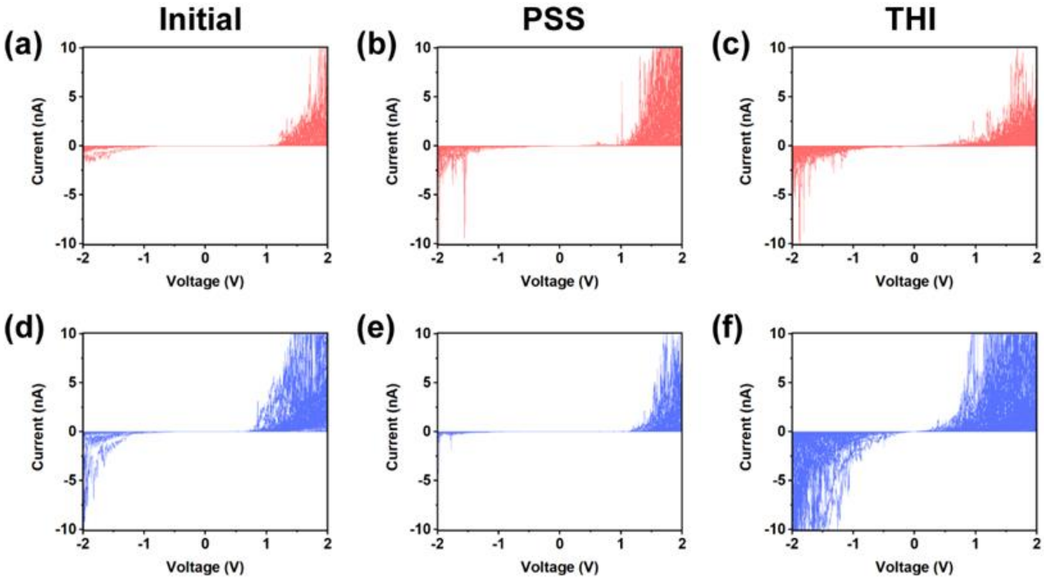

Figure S35. I-V curves (raw data) for the (*S*,*S*)-(*M*,*M*)-*Z* layer absorbed on the Ti (10 nm)/Ni (120 nm)/Au (8 nm) substrate with the magnet pointing down (blue) or up (red). (a) and (d) present initial (*S*,*S*)-(*M*,*M*)-*Z*. (b) and (e) present irradiated state (*S*,*S*)-(*P*,*P*)-*E* (PSS). (c) and (f) present thermally relaxed (*S*,*S*)-(*M*,*M*)-*E* (THI).

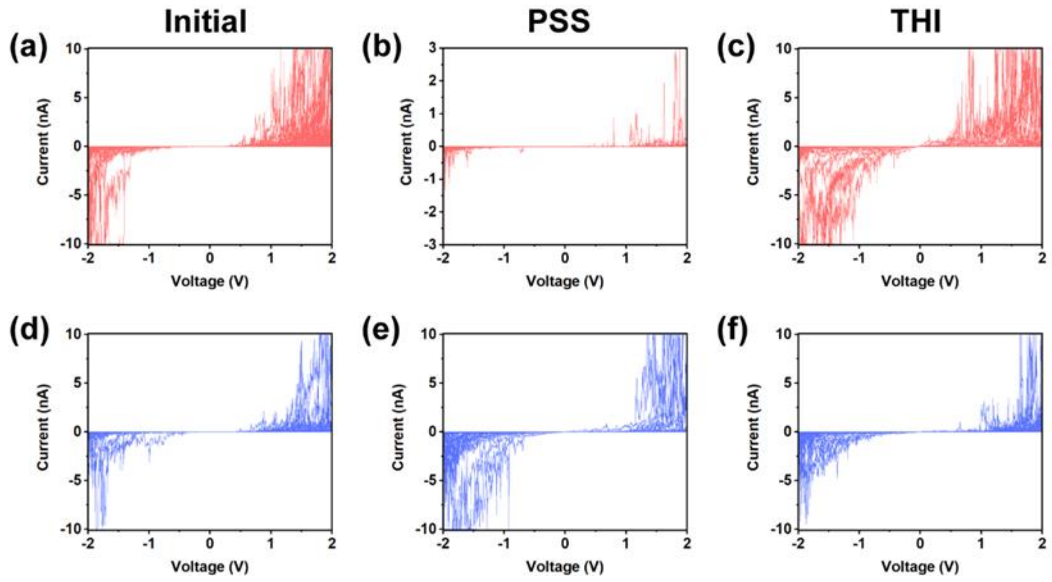

Figure S36. I-V curves (raw data) for the (*R*,*R*)-(*P*,*P*)-*E* layer absorbed on the Ti (10 nm)/Ni (120 nm)/Au (8 nm) substrate with the magnet pointing down (blue) or up (red). (a) and (d) present initial (*R*,*R*)-(*P*,*P*)-*E*. (b) and (e) present irradiated state (*R*,*R*)-(*M*,*M*)-*Z* (PSS). (c) and (f) present thermally relaxed (*R*,*R*)-(*P*,*P*)-*Z* (THI).

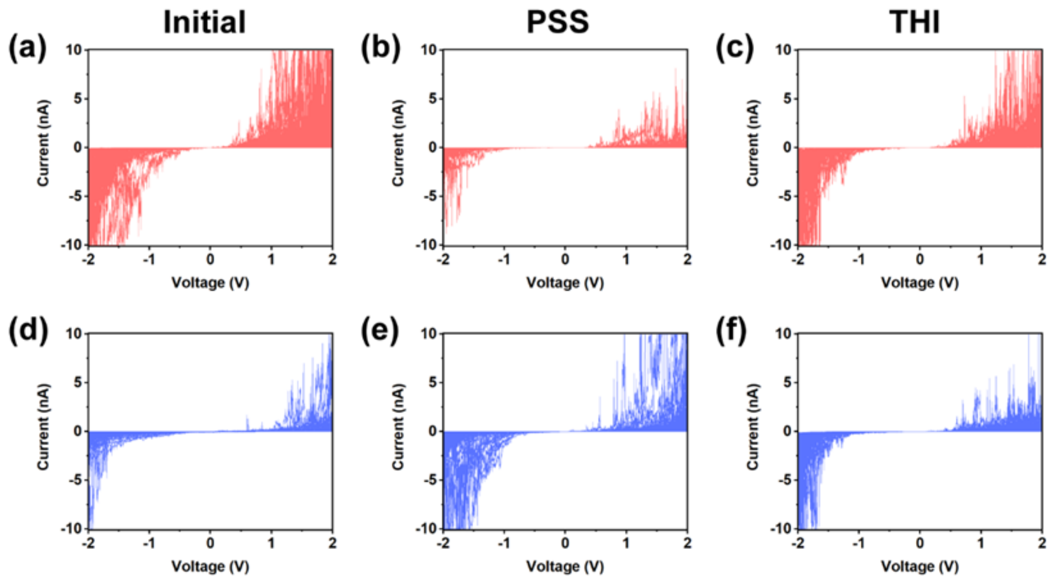

Figure S37. I-V curves (raw data) for the (*R*,*R*)-(*P*,*P*)-*Z* layer absorbed on the Ti (10 nm)/Ni (120 nm)/Au (8 nm) substrate with the magnet pointing down (blue) or up (red). (a) and (d) present initial (*R*,*R*)-(*P*,*P*)-*Z*. (b) and (e) present irradiated state (*R*,*R*)-(*M*,*M*)-*E* (PSS). (c) and (f) present thermally relaxed (*R*,*R*)-(*P*,*P*)-*E* (THI).

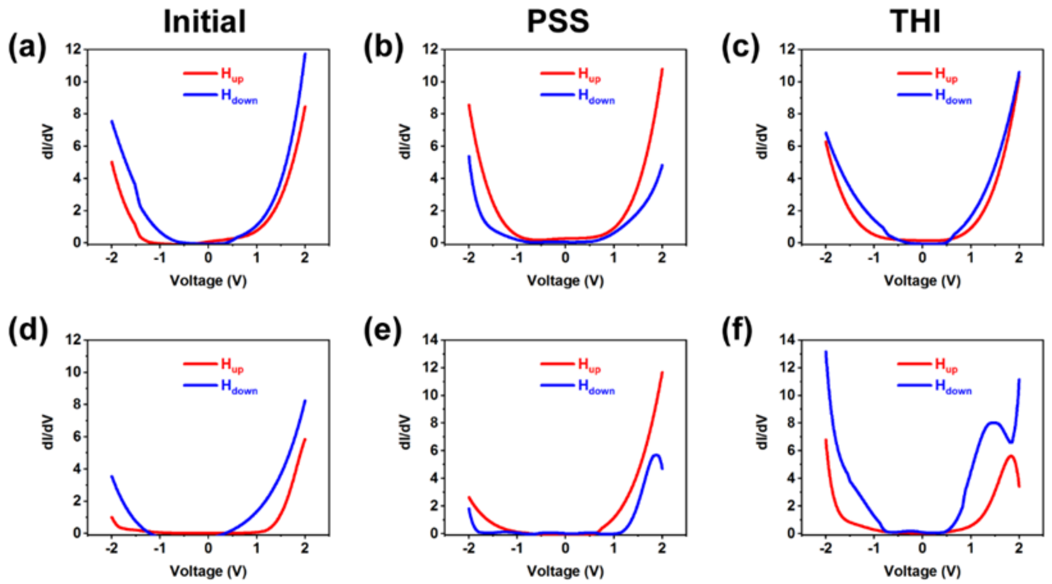

Figure S38. The density of states (dI/dV) computed from averaged I-V curves of (*S*,*S*)-(*M*,*M*)-*E* (**a** initial, **b** PSS, and **c** THI) and (*S*,*S*)-(*M*,*M*)-*Z* (**d** initial, **e** PSS and **f** THI) under magnetic field pointing up (red) or down (blue).

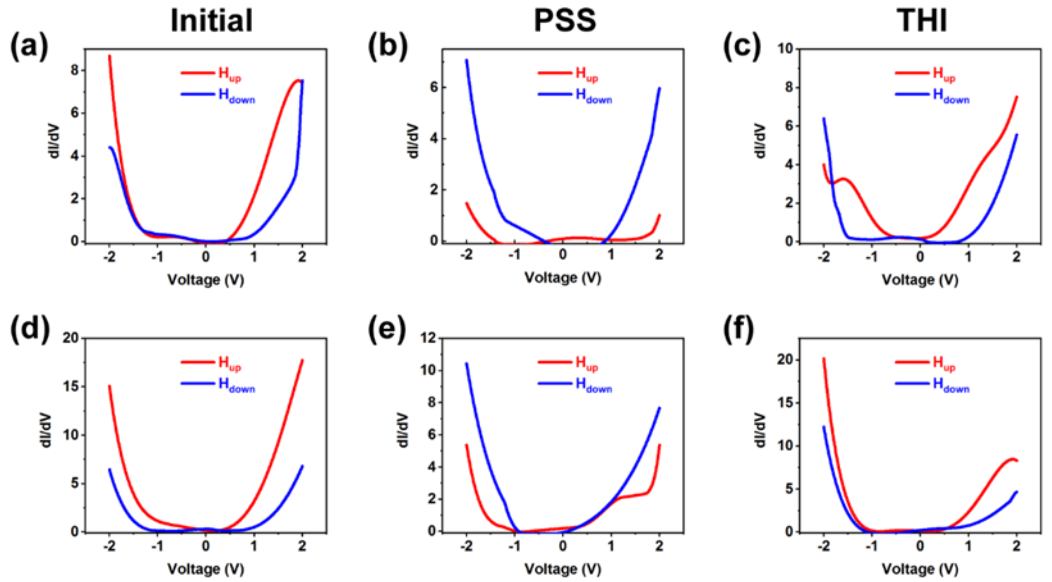

Figure S39. The density of states (dI/dV) computed from averaged I-V curves of (*R*,*R*)-(*P*,*P*)-*E* (**a** initial, **b** PSS, and **c** THI) and (*R*,*R*)-(*P*,*P*)-*Z* (**d** initial, **e** PSS and **f** THI) under magnetic field pointing up (red) or down (blue).

References

[1] T. van Leeuwen, J. Gan, J. C. M. Kistemaker, S. F. Pizzolato, M. C. Chang, B. L. Feringa, *Chem. Eur. J.* **2016**, *22*, 7054.
